# MAPPIN'SDM – The Multifocal Approach to Sharing in Shared Decision Making

**DOI:** 10.1371/journal.pone.0034849

**Published:** 2012-04-13

**Authors:** Jürgen Kasper, Frauke Hoffmann, Christoph Heesen, Sascha Köpke, Friedemann Geiger

**Affiliations:** 1 Faculty of Mathematics, Informatics, Unit of Health Sciences and Education, University of Hamburg, Hamburg, Germany; 2 Department of Primary Medical Care, University Medical Center Hamburg, Hamburg, Germany; 3 Unit of Health Sciences and Education, University of Hamburg, Hamburg, Germany; 4 Institute of Neuroimmunology and Clinical MS Research (INiMS), University Medical Center Hamburg, Hamburg, Germany; 5 Nursing Research Group, Institute of Social Medicine, University of Lübeck, Lübeck, Germany; 6 Tumor Center, University Medical Center Schleswig-Holstein, Kiel, Germany; 7 Department of Pediatrics, University Medical Center Schleswig-Holstein, Kiel, Germany; Innsbruck Medical University, Austria

## Abstract

**Background:**

The wide scale permeation of health care by the shared decision making concept (SDM) reflects its relevance and advanced stage of development. An increasing number of studies evaluating the efficacy of SDM use instruments based on various sub-constructs administered from different viewpoints. However, as the concept has never been captured in operable core definition it is quite difficult to link these parts of evidence.

This study aims at investigating interrelations of SDM indicators administered from different perspectives.

**Method:**

A comprehensive inventory was developed mapping judgements from different perspectives (observer, doctor, patient) and constructs (behavior, perception) referring to three units (doctor, patient, doctor-patient-dyad) and an identical set of SDM-indicators. The inventory adopted the existing approaches, but added additional observer foci (patient and doctor-patient-dyad) and relevant indicators hitherto neglected by existing instruments. The complete inventory comprising a doctor-patient-questionnaire and an observer-instrument was applied to 40 decision consultations from 10 physicians from different medical fields. Convergent validities were calculated on the basis of Pearson correlation coefficients.

**Results:**

Reliabilities for all scales were high to excellent. No correlations were found between observer and patients or physicians neither for means nor for single items. Judgements of doctors and patients were moderately related. Correlations between the observer scales and within the subjective perspectives were high. Inter-perspective agreement was not related to SDM performance or patient activity.

**Conclusion:**

The study demonstrates the contribution to involvement made by each of the relevant perspectives and emphasizes the need for an inter-subjective approach regarding SDM measurement.

## Introduction

Currently, there is broad consensus in the literature on health policy that shared decision making (SDM) represents the best practice model for medical decisions [1]. In the last decade, considerable growth of SDM has been recognized not only as regards the body of literature or the number of studies referring to SDM, but also regarding the concept itself and the scope of application of the term SDM [2].

Initially, SDM was introduced as a relatively narrowly defined communication method constituted by few criteria referring to a democratic style of communication between health professionals and the patients mutually involved in decision making [3], [4].

In recent years the term SDM underwent progressive proliferation. Instead of seeking an operationalization of the core construct, i.e. the *two way exchange of information* within a doctor-patient-dyad [3], most efforts were undertaken to make the idea transferable to broader health care contexts.

Nowadays there is nearly no area in health care which does not to some respect refer to SDM. SDM is referred to on the micro, meso and macro level of health care systems [5]. Beyond the process of decision making itself, SDM is applied to the decision context and also to the issue of outcomes of a decision [6].

This wide scale permeation of health care by SDM reflects the concept's relevance and advanced stage of development [7]. However, it could be argued that the concept is jeopardized to sustain a loss of power by its diffusion even before it has been basically understood [8]. Evaluation of SDM as applied in very different ways to various health care contexts can hardly yield evidence on its efficacy and appropriateness in general, but rather on partial aspects of the construct. However, as this has never been captured in a clear and operable core definition [2], [9], it remains difficult to link these different parts of evidence. It is not yet understood why some SDM-interventions, e.g. patient decision aids, effectively impact on the quality of decisions and others do not [10], [11]. Therefore, a need for theoretic foundation of SDM interventions regarding the mechanisms by which effects are mediated has been claimed [8], [12]. Appraisal of efficacy of SDM aiming to promote medical decision making closely depends on the quality of measurement methods. Reviewing methods to assess SDM has been considered difficult, since existing instruments address a wide variety of constructs which they approach from different viewpoints [6]. Recent reviews conclude that most existing instruments lack sufficient validation [5], [6], [13], [14]. Some instruments are constructed as observer scales focussing physicians' behavior [15]–[19]. Others are to be administered by patients or physicians assessing their perception of involvement [20], [21]. However, there still is a considerable number of omissions among operationalized perspectives [6]. E.g. the observer's focus on the patient and on the dyad as a unit have not yet been operationalized. Moreover, a theoretic framework is missing mapping the different SDM-measures regarding their specific perspectives, constructs and measurement units. This would allow for a better understanding and classifying results of SDM intervention studies [6]. Even within the sub-group of SDM instruments aiming to assess patient involvement in a narrow sense, studies indicate a broad variety of constructs. Pronounced discrepancies can be found between measures when assessing communication from different viewpoints (physician, patient, observer) [6], [22]. No correlations between an objective observer's and patients' ratings were found in a study investigating patient involvement within 76 consultations negotiating treatment decisions in multiple sclerosis [22]. Similar results have been reported from other studies [15], [23]–[25]. Judgements on patient involvement have been shown to be incongruent not only between observers and patients but also between observers and physicians and between patients and physicians and finally to considerable extent between different measures administered by the same patients [14], [23], [26]–[33]. However, the instruments used in these comparisons slightly differed regarding their specific selection of SDM indicators. Therefore, the adjusted degree of incongruence between perspectives from which SDM assessments are administered cannot conclusively be estimated [22]. Since knowledge on how the different parties' perceptions of the communication are interrelated might be crucial to define what constitutes involvement, there is a need to systematically investigate measurements on an inter-subjective level to receive a full picture of the communication [22].

The current study aimed at investigating interrelations of SDM indicators administered from different perspectives. In contrast to earlier studies assessing decision-making situations with different SDM measures [14], [15], [23]–[24], [27], this study used a systematic approach mapping judgements from different perspectives and constructs referring to an identical set of SDM indicators. Therefore, a comprehensive inventory called MAPPIN'SDM (Multifocal Approach to the Sharing in SDM) was developed. As a framework, MAPPIN'SDM categorizes all existing measurement approaches and makes them comparable on an empirical basis. The study aimed to eliminate possible sources of incongruence between existing SDM measures rather than to introduce a new SDM measure or to demonstrate contributions of each of the relevant perspectives (table 1).

**Table 1:** MAPPIN'SDM overview.

MAPPIN-Focus	Perspective	Instrument	Construct	Unit	
**1 Obs_doctor_**	**the observer's perspective on doctor's SDM behavior**	**observer**	**observation instrument**	**behavior**	**doctor**
**2 Obs_patient_**	**the observer's perspective on patient's SDM behavior**				**patient**
**3 Obs_dyad_**	**the observer's perspective both parties' (as a unit) SDM behavior**				**dyad**
**4 Qdoc_dyad(b)_**	**the doctor's perspective on SDM behavior**	**doctor**	**questionnaire**	**behavior**	**dyad**
**5 Qdoc_dyad(r)_**	**the doctor's perception of SDM**			**result**	
**-**			not operationalized	behavior	patient
**-**				result	
**6 Qpat_dyad(b)_**	**the patient's perspective on SDM behavior**	**patient**	**questionnaire**	**behavior**	**dyad**
**7 Qpat_dyad(r)_**	**the patient's perception of SDM**			**result**	
**-**			not operationalized	behavior	doctor
**-**				result	

The table illustrates the organization of the MAPPIN'SDM inventory by indicating the constituting elements for the seven foci of measurement. Each of which represents a separate view on the communication and is supposed to apply the identical set of 15 SDM indicators.

### The multifocal approach

In the following, the concepts architecture is explained unfolding the definitions of six constituting elements (underscored): the three perspectives, two constructs, three units, and seven foci which result in a set of three instruments, each assessing the same 15 indicators (table 1).

### Three perspectives

MAPPIN'SDM includes all three perspectives relevant to SDM measurement, referring to the different viewpoints from which communication is judged (physician, patient, observer).

### Two constructs

To allow for comparison of observation based judgements by observers and subjective judgements by physicians and patients, the inventory includes the two fundamentally different ways to conceive the construct *involvement*. *Construct* in this context refers to the subject of measurement. The first construct “behavior" is usually underpinning the observation based SDM-instruments. It can be defined as behaviors attempting to involve the two parties in the decision-making process. Here, the crucial question is: “Does the doctor or the patient undertake efforts to make the particular SDM issue explicit (and by doing so involve each other in the communication)?". The second construct “result" which is not accessible by observation is the extent of actual involvement achieved. It is the perceived (communication-) result in terms of SDM. Here, the crucial question is: “Did you feel involved in the communication (on e.g. the pros and cons of the available options) during the consultation?" For example, the SDM indicator “*listing of available options*" is shaped differently by the two constructs: (1) To assess *behavior*, the appropriate question would be: “*Were the options listed?*" whereas (2) to assess the *result*, it would be: “*Do I now know my options*?". While the observer can only judge the communication (mediated) based on the first construct, parties involved in the communication can judge both constructs.

### Three units

MAPPIN'SDM also addresses the three relevant units of measurement. ‘Unit’ in this context refers to the (social) object upon which the measurements are made. Existing instruments commonly address the physician as unit of measurement, by focussing on whether the physician initiates and displays actions indicating SDM [6], [14]–[16]. This unilateral focus indicates that the doctor alone should take the responsibility for the communication process and should therefore control it. Comparable to the “Perceived Involvement in Care Scale" (PICS, [34]), MAPPIN'SDM also focuses on the patient, as in our understanding, also the patient could – and ideally should – be initiator of involvement. Moreover, as a third unit, the dyad is considered, integrating doctor and patient. These arrangements were made to allow for investigating research questions based on the assumption that the quality of a decision does not solely depend on whether certain aspects are brought up. In contrast, it is of equal importance, which of the parties brings up a specific aspect or to what extent both parties participate in the discussion of individual aspects. As a consequence, full assessment of the process necessitates consideration of both dyad members individually and as a unit.

### Seven foci

These considerations result in seven foci (table 1). Focus in this context is defined as the lowest common denominator of perspective, construct and unit. The construct *behavior* can be defined for three different perspectives (observer, doctor, patient) and also for three different units (doctor, patient, dyad), while the construct, *result*, can be defined for two perspectives (doctor, patient) and two units (doctor, patient). Among the existing instruments, focus 1 (observation of doctor) is well known from existing observer scales such as the OPTION scale [16] or the Rochester decision making scale [15], while foci 2 and 3 (observation of patient and dyad) have hitherto not been realized.

Foci 4 and 6, addressing “SDM behavior" as observed by doctor and patient have not been acknowledged so far since both patients and physicians can more easily respond to questions focusing on their perception of the “SDM result" (foci 5 and 7). However, these two foci were operationalized to provide opportunity to interrelate observers' and patients' or doctors' judgements based on identical constructs. Strictly speaking, complete variation of the system would lead to another four foci (patients and doctors judging each other's behavior and perception) which were not realized in the MAPPIN'SDM inventory since measurement of units and constructs by crossing over perspectives seemed rather complicated and was not required by our specific research questions. In summary, the inventory comprises three observer ( = Obs) foci and four self-administered foci (questionnaire = Q), made up of two constructs (behavior = b and result = r) and two subjective parties (doctor = doc and patient = pat), leading to the following seven scales (abbreviations): Obs_doctor_, Obs_patient_, Obs_dyad_, and Qdoc_dyad(b)_, Qdoc_dyad(r)_, Qpat_dyad(b)_, Qpat_dyad(r)_ (table 1).

### Two instruments

The seven foci of measurement are operationalized in two instruments, one of which is to be used as an observation based instrument (ideally based on video documents), the other one, a questionnaire, to be administered by patients and physicians (ideally directly after a consultation) (Appendix S1, S2, S3). Analogous to the OPTION scale, observation as well as questionnaire items are presented as statements [16]. The extent to which the given indicator is performed has to be judged on five point Likert scales. A manual was developed providing comprehensive instructions how to use the observation instrument (Appendix S4). To assess comprehensibility of the questionnaire which is used without a manual, the questionnaire items were piloted with 10 patients and physicians each. This process led to stepwise revision of item wording to optimize understanding while keeping it close to observer items. If necessary, an additional explanation or an example was supplemented. Participants of the piloting groups considered the set of 15 indicators relevant and exhaustive. The two questionnaires for doctors and patients are identical apart from adjustments of personal pronouns. Reliability of the observer tool was tested in a pre-study re-analysing an existing pool of 76 videos of consultations on multiple sclerosis treatment decisions [35]. Inter-rater-reliability was high to excellent in all three scales (Obs_doctor_ = .90, Obs_patient_ = .85, Obs_dyad_ = .91). After scoring, the set of 15 indicators was considered exhaustive by the experienced observers [35].

### Fifteen SDM indicators

All seven foci are based on an identical set of 15 aspects which we considered essential to indicate SDM (table 2). When defining the set of indicators, we started from the set of 12 indicators in the OPTION scale [36]. All indicators were adopted keeping their basic idea and wording, as far as possible and appropriate for the seven foci. An authorized German translation already existed [36], but, based on theoretic, language or communication considerations, some items had to be slightly refined to better fit the basics of the SDM concept. Two items had to be fused for methodological reasons [9]. Four new indicators were included (described in more detail in [35]): Firstly, rather than being just a concept for organizing a dialogue, the SDM concept is also concerned with information quality. In contrary to existing instruments, the set of MAPPIN'SDM indicators therefore considers criteria of evidence based patient information (EBPI [37]). One indicator hence was defined dealing with the issue of referring to the source of any information or recommendation given (Item 8). Moreover, the appraisal of existing indicators – such as ‘communicating risks and side-effects of each option’ (Item 6) was specified according to EBPI criteria. Secondly, the issue of checking patients' understanding was supplemented by an indicator considering whether the doctor has understood the patient's viewpoint correctly (Item 10). Thirdly, corresponding to the already existing indicator focussing on opportunities for the patient to ask questions a new indicator was added defining opportunities to ask questions or express uncertainties given to the doctor (Item 12). Fourthly, based on empirical findings form analysing previous decision videos [22], another new indicator defined an additional competency of meta-communication about decision-making strategies (Item 13).

**Table 2:** Comparison of OPTION and MAPPIN'SDM.

SDM aspect	OPTION item no.	MAPPIN'SDM item no.
Defining problem	1	1
Equipoise statement	2	2
Preferred communication approach	3	3
Listing options	4	5
Pros & cons	5	6
Expectations	6	7
Worries	7	
Indicating source of recommendations/evidence		8
Doctor's evaluation of patient's understanding	8	9
Patient's evaluation of physician's understanding		10
Opportunity for questions (from patient)	9	11
Opportunity for questions (from physician)		12
Role attribution	10	4
Supporting strategies of decision-making		13
Indicate decision	11	14
Follow up arrangements	12	15

Set of indicators of shared decision making of MAPPIN'SDM compared to that of the OPTION scale.

### Hypotheses

Due to the explorative character of the research question, the study did not apply specific hypotheses about the degree of inter-relatedness of perspectives and foci. 1) However, in view of our literature review, we expected the degree of congruence between different measurement perspectives (between-perspective-correlations) to be very limited 2) As a proof of the SDM concept's basic idea, we expected doctor- and patient activity as assessed by the observer to be positively correlated (within perspective correlation). 3) We expected patients and doctors with higher skill levels to be better observers and, therefore, their performance to be associated with inter-perspective agreement (impact of performance). 4) Since the dyad's performance also reflects patient activity we expected higher patient activity to reduce (within-observer-) correlation between doctor and dyad (impact of performance). 5) We assumed the parties respond congruently to the different constructs, behaviour and result. Therefore, we expected high (within-party-) correlations between foci 4&5 and between foci 6&7 respectively. 6) We selected three indicators, considered most essential for patients' involvement [13], to test the same correlations on item level (Indicator 2: *Equipoise*, indicator 6: *Communication of risks*, indicator 14: *Agreeing on a decision*)

## Methods

### Ethics statement

The study protocol was approved by the ethics committee of the University Medical Center Kiel, Germany; and all participants gave written informed consent for record, analyses and publication of their data collected within this study.

### Design

The study was designed as a survey assessing SDM within doctor-patient consultations including a medical decision within different medical disciplines. Observations were made of physicians taking part in a SDM training program.

Our own results as well as results reported by other authors had always shown relatively low SDM performance levels of physician behaviors [16], [22], [38], [39]. To allow for sufficient variance regarding physicians' skill levels, we provided SDM training to participating physicians. The training comprised (1) a SDM-manual explaining the communication background and providing detailed examples for all 15 SDM-indicators, (2) a video tutorial, presenting examples for all 15 SDM indicators drawn from different doctor-patient consultations and (3) a face to face feedback referring to one of the participants' consultations documented on video. Each participating physician was asked to record a sequence of each of four consultations representing four training levels. Training component (1) was provided after the baseline consultation, (2) after the first level, (3) after the second level consultation. Patients participated in the study only once.

### Participants and recruitment

Despite the explorative character of the study, the sample size should allow for determining reliable values for correlations. In total, 40 doctor-patient consultations were recorded in the Hamburg University Medical Center with 40 patients and 10 physicians, four from the multiple sclerosis out-patient department, three from the department of dental medicine, and three general practitioners working in private practice in and around Hamburg. While physicians were contacted directly, patients were recruited by participating physicians.

### Data collection

The MAPPIN'SDM inventory was applied in full to each appointment. Consultations were documented using video recordings and MAPPIN'SDM questionnaires completed by doctor and patient. The doctor-patient questionnaire included 30 items each (doctor: Qdoc_dyad(b)_ and _(r)_, patient: Qpat_dyad(b)_ and _(r)_) (Appendix S2, S3). To compensate for varying degrees of familiarity with SDM-indicators, doctors and patients were instructed to read the MAPPIN'SDM-items before the consultation. After agreeing on a reference decision indicated on the first page, the questionnaires were filled in immediately after the consultation, independently by doctor and patient.

### Data analysis

Within each video, time markers were set indicating the entire decision making process based on corresponding statement made by doctor and patient in the questionnaire. Videos were analysed in random order by three trained raters with previously proven high inter-rater-reliability, one of them coding all 40 videos, the other two coding 20 consultations each. Inter-rater-reliability was calculated between rater pairs based on arithmetic mean scale values using Pearson correlation coefficients. Inter-scale correlations were built based on arithmetic means of both raters' mean scale values using Pearson correlation coefficients. Values could range from 0 (*poor performance*) to 4 (*excellent performance*). By use of a pseudonym, raters were blinded towards questionnaire data and the doctors' level of SDM training. Levels of higher and lower performance and of more or less patient activity were defined by median split. Differences between correlations were checked for statistical significance with Fisher's Z test. Data were processed and analyzed using SPSS version 16.

## Results

### Details about consultations (Table 3)

**Table 3:** Consultation sample.

	specialisation	sex		decision topic	medical problem	Length (min:sec)
		Doc.	Pat.			
1	Neurologist	♂	♂	liquor diagnostic	Suspected MS	24:19
			♂	immunotherapy	MS	23:56
			♂	immunotherapy	MS	19:47
			♀	immunotherapy	MS	16:25
2	Neurologist	♀	♀	immunotherapy	MS	14:40
			♀	immunotherapy	MS	08:45
			♀	diagnostic	Suspected MS	10:08
			♂	immunotherapy	MS	10:29
3	Neurologist	♀	♀	immunotherapy	MS	05:44
			♀	immunotherapy	MS	19:47
			♀	immunotherapy	MS	05:20
			♀	immunotherapy	MS	09:30
4	Neurologist	♂	♀	immunotherapy	MS	38:45
			♀	immunotherapy	MS	24:40
			♀	immunotherapy	MS	16:16
			♂	immunotherapy	MS	31:13
5	Dentist	♂	♀	dental prostheses	Tooth space	24:11
			♂	treatment	Limited mouth opening	28:22
			♀	dental crown	Caries	09:43
			♀	dental prostheses	Tooth space	17:29
6	Dentist	♂	♀	dental prostheses	Tooth space	16:56
			♂	dental prostheses	Edentulism	05:53
			♂	Crown material	Caries	07:12
			♂	Implant or bridge	Tooth space	22:50
7	Dentist	♂	♀	dental prostheses	Edentulism	15:35
			♂	dental prostheses	Edentulism	40:36
			♂	dental prostheses	Tooth space	16:00
			♂	Dental filling	Caries	19:43
8	Internist	♂	♂	treatment	Hypertension	17:23
			♂	treatment	Hypertension	14:36
			♂	risk prophylaxis	Diabetes	19:01
			♀	treatment	Hypertension	14:38
9	Internist	♀	♂	Diagnostic	Abdominal pain	02:24
			♀	Diagnostic	Chronic anaemia	03:36
			♀	drug treatment	Fibromyalgia	02:42
			♂	surgical treatment	Carpal tunnel syndrome	03:57
10	GP	♂	♂	prophylaxis	Swine flu	04:43
			♂	prophylaxis	Swine flu	13:20
			♂	prophylaxis	Swine flu	11:20
			♂	prophylaxis	Swine flu	11:50

MS = multiple sclerosis, GP = general practitioner.

Among the 40 patients participating in the study, 22 were male. The 10 physicians (7 male) were specialists in neurology, dental and internal as well as general medicine. A wide range of medical topics were issued within the decision making consultations. The length of consultations ranged from 2.5 to 51 minutes (mean 19.5 min), the lengths of decision sequences from 2.5 to 38.8 minutes (mean 15 min).

### Reliabilities and scale properties

Inter-rater-reliabilities were high to excellent in the observer scales (Obs_doctor_: r = .87, Obs_patient_: r = .81, Obs_dyad_: r = .74). Internal consistencies of the four questionnaire scales were high (Cronbachs alpha: Qdoc_dyad(b)_ = .91, Qdoc_dyad(r)_ = .94, Qpat_dyad(b)_ = .92, Qpat_dyad(r)_ = .94). Judgements of observers scored low to medium (Obs_doctor_ = 1.2(SD = .4), Obs_patient_ = 0.7(SD = .3), Obs_dyad_ = 1.4(SD = .4), while subjective judgements were high (Qdoc_dyad(b)_ = 2.5(SD = .7), Qdoc_dyad(r)_ = 2.9(SD = .6), Qpat_dyad(b)_ = 2.7(SD = .7), Qpat_dyad(r)_ = 3.2(SD = .6)).

### Between-perspective-correlation (hypothesis 1)(Table 4)

**Table 4:** Inter-relations of MAPPIN'SDM foci.

	Obs_patient_	Obs_dyad_	Qdoc_dyad(b)_	Qdoc_dyad(r)_	Qpat_dyad(b)_	Qpat_dyad(r)_
Obs_doctor_	r = .64(<.001)	r = .96(<.001)				
Obs_patient_		r = .80(<.001)				
Obs_dyad_			r = .14(.4)		r = −.22(.19)	
Qdoc_dyad(b)_				r = .85(<.001)	r = .45(.004)	
Qdoc_dyad(r)_						r = .37(.02)
Qpat_dyad(b)_						r = .81(<.001)
Qpat_dyad(r)_						

The table shows Pearson correlation coefficients of pairwise related judgements by MAPPIN'SDM different measurement foci. Abbreviations are explained in detail in table 1.

Observers' judgements were not interrelated with subjective judgements (r(Obs_dyad_;Qdoc_dyad(b)_) = .14, p = .4; r(Obs_dyad_;Qpat_dyad(b)_) = −.22, p = .19). However, moderate correlations were shown between doctors' and patients' judgements of SDM-behavior and between both parties' perception of involvement (r(Qdoc_dyad(b)_;Qpat_dyad(b)_) = .45, p = .004; r(Qdoc_dyad(r)_;Qpat_dyad(r)_) = .37, p = .02).

### Within-perspective-correlation (hypotheses 2 and 5)

SDM behavior as observed by subjective parties was highly correlated with the corresponding level of perceived SDM (result) (r(Qdoc_dyad(b)_;Qdoc_dyad(r)_) = .85, p<.001; r(Qpat_dyad(b)_;Qpat_dyad(r)_) = .81, p<.001). Observers' judgements on the three foci were moderately or highly inter-related (r(Obs_doctor_;Obs_patient)_ = .64, p<.001; r(Obs_doctor_;Obs_dyad_) = .96, p<.001 r(Obs_patient_;Obs_dyad_) = .80, p<.001).

### Impact of performance level on inter-relatedness of perspectives (hypotheses 3 and 4)

SDM performance as defined by observers' rating of the dyad did not impact on the inter-relatedness of MAPPIN'SDM measurement perspectives (r_high_(Obs_dyad_;Qdoc_dyad(b)_) = −.03; r_low_(Obs_dyad_;Qdoc_dyad(b)_) = −.02, p = .97; r_high_(Obs_dyad_;Qpat_dyad(b)_) = −.3; r_low_(Obs_dyad_;Qpat_dyad(b)_) = −.2, p = .63). Accordingly, patient activity in terms of SDM as defined by observers did not impact on inter-relatedness of different perspectives on the communication (r_high_(Obs_dyad_;Qdoc_dyad(b)_) = −.08; (r_low_(Obs_dyad_;Qdoc_dyad(b)_) = .06, p = .69; (r_high_(Obs_dyad_;Qpat_dyad(b)_) = −.16; (r_low_(Obs_dyad_;Qpat_dyad(b)_) = .11, p = .43; (r_high_(Qdoc_dyad(r)_;Qpat_dyad(r)_) = .46; (r_low_(Qdoc_dyad(r)_;Qpat_dyad(r)_) = .40, p = .83). In contrary to our assumption (hypothesis 4) patient activity also did not impact on the correlation of observer judgements for doctor and dyad (r_high_(Obs_doc_;Obs_dyad_) = .97; (r_low_(Obs_doctor_;Obs_dyad_) = .93, p = .19).

### Inter-relatedness on item level (hypothesis 6)

Inter-relatedness between MAPPIN'SDM measurement perspectives of three selected SDM-indicators (Indicator 2: *Equipoise*, indicator 6: *Communication of risks*, indicator 14: *Agreeing on a decision*) did not differ significantly from results reported above for mean score levels.

## Discussion

The study attempted to compare different relevant perspectives on patient involvement using MAPPIN'SDM, a comprehensive system including all existing measurement approaches and a homogenized set of SDM indicators. To account for the full picture of SDM, the inventory partly had to supplement hitherto lacking pieces of the puzzle. In particular, two additional observer foci (patient and dyad) and additional indicators were defined. For purpose of comparability with observer data, subjective perspectives (patient, doctor) were defined for both constructs i.e. the observation of behaviour and the immediate perception of SDM (result).

### Principal results

Our study found judgements regarding patient involvement in decision making processes from any of the observers' foci (on doctor, patient or the dyad) completely unrelated to the subjective reporting of patients and doctors. Judgements of patients and physicians were moderately correlated. The same picture was exemplarily found on item level, for three most crucial indicators (Equipoise, Risk communication, Agreeing on a decision). Within-perspective-correlations were high, e.g. between the three observer foci and between each of the parties' report of behavior and perception of involvement. Inter-relatedness of the three perspectives on SDM was not influenced by either the dyad's degree of SDM performance or the extent of patients' activity in initiating SDM indicators.

### Limitations of the study

Generalizability of our results on interrelatedness of judgement perspectives and on the potential impact of the skill level on these interrelations is limited with regard to the moderate to low performance of doctors and patients. In particular, an impact of patient activity on the congruence of perceiving the communication seems still likely, if patients achieve considerably higher levels of active involvement. With regard to the high efforts of assessment and analysis of the communication measures and the explorative character of the study, the consultation samples and even more the physician sample were small. Although medical topics and specializations varied, results cannot be deemed as representative for doctor-patient-dyads in general or for any particular sub-population. Our results, therefore, have to be regarded with caution as to our knowledge it has not been conclusively shown that dynamics of doctor-patient-dyads varying with setting and medical subject are not relevant to the measurement of patient involvement.

Due to the artificial setting with presence of a camera, doctors' behavior during the study might have been non-representative and biased e.g. in terms of social desirability. This is a general and hitherto unsolved problem regarding the validity of observation data. Simulated patients acting incognito would yield more natural behavior examples. However, such methods would imply more sophisticated ethical and technical considerations.

For practical reasons, patients and consultations were selected by the doctors. Although a clear inclusion criterion was given, doctors might have biased their selection following own priorities and beliefs about which kind of patient or topic would be most suitable for the study. Positive-selection of this kind could have led to overestimation of doctors' skills, which does not seem a problem in our study.

Piloting of items before and arrangements made during the study might not have been sufficient to adjust level of familiarity with the given items and concepts among the three perspectives. Although this imbalance might have to some extent amplified the diversity of the concepts, it seems inevitable and inherent within the perspectives. Providing the quality criteria to the parties before the consultation might on the other hand have led to some artificial efforts to adhere to these criteria on both sides. A potential learning effect in this regard, however, does not challenge our conclusions, which were about interrelatedness of judgments rather than about absolute level of SDM.

In our view, a serious limitation of the measurement approach used, lies in the equal weighting of indicators in SDM analyses. Emphasis of certain indicators of higher relevance seems reasonable and should be issued in further studies. However, an introduction and evaluation of a new weighting key would have led to further complexity in this study.

It may be argued that adjusting the alpha level for multiple testing of correlations would have been appropriate to avoid false positive correlations. However, as in this study there were hardly any significant correlations, adjustment would not have changed our main conclusions.

### Results in context

Although other studies have suggested similar results of incongruence between existing measurement perspectives [15], [22]–[24], the present results attain pronounced emphasis with respect to the endeavours undertaken to homogenize measurements. As incongruence appears unabated also in this study and measurement difficulties become increasingly unlikely as an explanation for this phenomenon, the present study considerably adds to the knowledge that the three perspectives are anything but redundant.

We want to point out two important implications:

Firstly, a unilateral approach to SDM using one of the perspectives as a proxy for the full picture is misleading, because further perspectives probably yield different results. Conclusions that can be drawn from any of such measurements are limited to the ability of the single perspectives to recognize SDM. For instance, data evaluating a SDM training indicating better skills of the doctors do not necessarily indicate a change in the patient-relevant communication. It might be argued that such incongruence only reflects the concept's complexity and that SDM has to be regarded as divided into sub-constructs such as the subjective perceptions of or the attitudes towards SDM [6]. A variety of concepts might underpin the perception of the same issues such as e.g., *whether expectations and worries of the patient had been sufficiently explored*. This would mean that beyond measurement issues, SDM as judged by observers is (and possibly should be) something different compared to SDM as perceived by patients or physicians [22]. As mentioned above, the SDM literature seems to reflect this proliferation of the idea and the concept. As a consequence of this dynamics, discussion about SDM seems increasingly complicated by the difficulty to clearly agree on the level, the context, the process, and the construct which is referred to in a particular case. However, the SDM core construct is defined by explicitness regarding the information process and by inter-subjectivity regarding the inter-personal actions in a decision making process [3], [9]. If SDM as determined by an observer watching doctors' behavior is not reaching patients' perception, it cannot be considered SDM.

Secondly, non-redundancy of the different perspectives' judgements on SDM means that each of the perspectives considerably contributes to the definition of the construct. As it would not be reasonable to award the power of definition to one of the perspectives alone [22], all have to be regarded as essential. If this finding drawn from a rigorous methodological approach seems familiar and easy to accept, this might be due to the reader's awareness of the concept's basic assumptions [3]. Following these, parties negotiate the decision subject and mutually approximate an agreement reflecting a shared definition of what can be seen as the best choice in an individual case [9]. Since this process is intended to refer to criteria of evidence based patient information rather than realizing a democratic discourse on any possible content, the observer's own expertise is essential too. Therefore, a measure of patient involvement is required to integrate these perspectives. This can be realized by developing compound measures including the three relevant perspectives and defining SDM on the level of inter-relatedness. Accordingly, a coefficient for SDM has been suggested including observers' and patients' judgements and additionally a measure of concordance between patient and doctor [40]. However, as triadic SDM measurement might mostly overstrain existing resources, this complex approach should only be used in research aiming at deriving measures more easy to administer. One possibility would be to define SDM on the level of certain markers with a proven agreement with the more complex reference system. However, such indicators have not yet been identified.

Recent approaches to SDM measurement are increasingly considering the interpersonal character of the core construct by employing corresponding measures on both sides of the dyad [5], [6], [41], [42]. This method allows for investigation of the interpersonal relation regarding the perception of the particular construct. However, there is still no strong theoretical basis about how a dyadic or triadic measure should turn out to indicate SDM.

### Conclusion

The importance to consider and combine all perspectives in SDM measurement has been shown as a result of pronounced non-redundancy of judgements on SDM from different perspectives using a systematic measurement approach (MAPPIN'SDM). This empirical result is in line with the core assumptions of the SDM concept. MAPPIN'SDM is a comprehensive and balanced approach covering all relevant perspectives on SDM and is suitable as an instrument to investigate the validity of existing measurement approaches.

## Supporting Information

Appendix S1 **MAPPIN'SDM observer sheet.** The observer sheet comprises the MAPPIN'SDM items used by observers coding the communication performance of doctors, patients and doctor-patient dyads. Scores have to be given for 15 items each based on observable behaviour. The observer sheet was developed in German language and is provided here as (based on retranslation) investigator authorized English language version.
(DOC)

Appendix S2 **MAPPIN'SDM (doctor-) questionnaire.** The MAPPIN'SDM questionnaire is supposed to be used by doctors assessing the communication quality in terms of SDM. The questionnaire comprises the same set of SDM indicators as the three foci of the MAPPIN'SDM observer instrument and the MAPPIN'SDM (patient-) questionnaire. In contrary to the observer instrument, scores have to be given based on subjective perception of the communication result rather than on behavioural attempts. The questionnaire was developed in German language and is provided here as (based on retranslation) investigator authorized English language version.
(DOC)

Appendix S3 **MAPPIN'SDM (patient-) questionnaire.** The MAPPIN'SDM questionnaire is supposed to be used by patients assessing the communication quality in terms of SDM. The questionnaire comprises the same set of SDM indicators as the three foci of the MAPPIN'SDM observer instrument and the MAPPIN'SDM (doctor-) questionnaire. In contrary to the observer instrument, scores have to be given based on subjective perception of the communication result rather than on behavioural attempts. The questionnaire was developed in German language and is provided here as (based on retranslation) investigator authorized English language version.
(DOC)

Appendix S4 **MAPPIN'SDM coder manual.** The MAPPIN'SDM coder manual provides comprehensive information and guidance for raters applying the MAPPIN'SDM approach. In particular, the manual comprises 64 pages and includes the following chapters: “Introduction", “Stage of research on SDM measurement", The MAPPIN'SDM method", “Using the manual", “The rater training", “Description of the indicators", “References", “Attachments". The coder manual was developed in German language and is provided here as investigator authorized English language version.
(DOC)
