# Correction: MAPPIN'SDM – The Multifocal Approach to Sharing in Shared Decision Making

**DOI:** 10.1371/annotation/3e489f03-e7e7-4b41-827e-caa85bb06466

**Published:** 2012-06-01

**Authors:** Jürgen Kasper, Frauke Hoffmann, Christoph Heesen, Sascha Köpke, Friedemann Geiger

There was an error in affiliation 3 for authors Jürgen Kasper and Frauke Hoffmann. Affiliation 3 should be: Institute for Communication in Medicine - Training and Research, Hamburg, Germany.

