# Supplementary material for: MAPPIN'SDM – The Multifocal Approach to Sharing in Shared Decision Making
Source: PLoS One. 2012 Apr 13;7(4):e34849. doi: 10.1371/journal.pone.0034849 (PMC3325952; doi:10.1371/journal.pone.0034849)
Supplement: Appendix S1 — MAPPIN'SDM observer sheet. The observer sheet comprises the MAPPIN'SDM items used by observers coding the communication performance of doctors, patients and doctor-patient dyads. Scores have to be given for 15 items each based on observable behaviour. The observer sheet was developed in German language and is provided here as (based on retranslation) investigator authorized English language version. (DOC) [file pone.0034849.s001.doc]

| **1** | The **clinician** draws attention to an identified problem as one that requires a decision-making process.  **MAPPIN’SDM observation sheet** | 0 | 1 | 2 | 3 | 4 |
| --- | --- | --- | --- | --- | --- | --- |
|  | The **patient** draws attention to his concrete problem as one that requires a decision-making process. | 0 | 1 | 2 | 3 | 4 |
|  | **Clinician and patient** agree on a concrete problem as one that requires a decision-making process. | 0 | 1 | 2 | 3 | 4 |

| **2** | The **clinician** states that there is more than one way to deal with the identified problem (‘*equipoise*’). | 0 | 1 | 2 | 3 | 4 |
| --- | --- | --- | --- | --- | --- | --- |
|  | The **patient** indicates that there is more than one way to deal with the concrete problem (‘*equipoise*’). | 0 | 1 | 2 | 3 | 4 |
|  | **Clinician and patient** discuss that there is more than one way to deal with the concrete problem (‘*equipoise*’). | 0 | 1 | 2 | 3 | 4 |

| **3** | The **clinician** ascertains the patient’s preferred approach to exchanging information (*e.g. in which setting, with which media, which time frame*). | 0 | 1 | 2 | 3 | 4 |
| --- | --- | --- | --- | --- | --- | --- |
|  | The **patient** participates in deciding on the preferred approach to exchanging information (*e.g. in which setting, with which media, which time frame*). | 0 | 1 | 2 | 3 | 4 |
|  | **Clinician and patient** choose an approach to exchanging information (*e.g. in which setting, with which media, which time frame*). | 0 | 1 | 2 | 3 | 4 |

| **4** | The **clinician** elicits the patient’s preferred level of involvement in decision-making. | 0 | 1 | 2 | 3 | 4 |
| --- | --- | --- | --- | --- | --- | --- |
|  | The **patient** expresses his preferred level of involvement in decision-making. | 0 | 1 | 2 | 3 | 4 |
|  | **Clinician and patient** discuss role distribution during the consultation. | 0 | 1 | 2 | 3 | 4 |

| **5** | The **clinician** lists the options (*If ‘doing nothing / deferring the decision’ is possible, this option should be included in the list*). | 0 | 1 | 2 | 3 | 4 |
| --- | --- | --- | --- | --- | --- | --- |
|  | The **patient** lists the options (*If ‘doing nothing / deferring the decision’ is possible, this option should be included in the list*). | 0 | 1 | 2 | 3 | 4 |
|  | **Clinician and patient** list the options (*If ‘doing nothing / deferring the decision’ is possible, this option should be included in the list*). | 0 | 1 | 2 | 3 | 4 |

**MAPPIN’SDM observation sheet**

| **6** | The **clinician** explains to the patient the pros and cons of the different options (*if applicable, also the pros and cons of ‘doing nothing’*). | 0 | 1 | 2 | 3 | 4 |
| --- | --- | --- | --- | --- | --- | --- |
|  | The **patient** discusses the pros and cons of the different options (*if applicable also the pros and cons of ‘doing nothing’*). | 0 | 1 | 2 | 3 | 4 |
|  | **Clinician and patient** weigh up the pros and cons of the different options (*if applicable, also the pros and cons of ‘doing nothing’*). | 0 | 1 | 2 | 3 | 4 |

| **7** | The **clinician** explores the patient’s expectations (*ideas*) and concerns (*fears*) about how to manage the concrete problem. | 0 | 1 | 2 | 3 | 4 |
| --- | --- | --- | --- | --- | --- | --- |
|  | The **patient** describes his expectations (*ideas*) and concerns (*fears*) about how to manage the concrete problem. | 0 | 1 | 2 | 3 | 4 |
|  | **Clinician** **and patient** discuss the patient’s expectations (*ideas*) and concerns (*fears*) about how to manage the concrete problem. | 0 | 1 | 2 | 3 | 4 |

| **8** | The **clinician** makes known to the patient the source upon which his information / recommendations are based (*scientific evidence, own judgement, preferences, conflicting interests*). | 0 | 1 | 2 | 3 | 4 |
| --- | --- | --- | --- | --- | --- | --- |
|  | The **patient** clarifies the source upon which medical information / recommendations are based (*scientific evidence, clinician’s judgement, preferences, conflicting interests*). | 0 | 1 | 2 | 3 | 4 |
|  | **Clinician and patient** clarify the source upon which medical information / recommendations are based (*scientific evidence, clinician’s judgement, preferences, conflicting interests*). | 0 | 1 | 2 | 3 | 4 |

| **9** | The **clinician** checks that the patient has understood the information. | 0 | 1 | 2 | 3 | 4 |
| --- | --- | --- | --- | --- | --- | --- |
|  | The **patient** clarifies how he understood the information given by the clinician. | 0 | 1 | 2 | 3 | 4 |
|  | **Clinician and patient** clarify whether the patient understood the information given by the clinician correctly. | 0 | 1 | 2 | 3 | 4 |

| **10** | The **clinician** makes sure that he has understood the patient’s viewpoint correctly. | 0 | 1 | 2 | 3 | 4 |
| --- | --- | --- | --- | --- | --- | --- |
|  | The **patient** makes sure that the clinician understands his viewpoint. | 0 | 1 | 2 | 3 | 4 |
|  | **Clinician and patient** clarify whether the clinician has understood the patient’s viewpoint correctly. | 0 | 1 | 2 | 3 | 4 |

**MAPPIN’SDM** observation sheet

| **11** | The **clinician** explicitly offers the patient opportunities to ask questions and to point out aspects he had not fully understood during the discussion. | 0 | 1 | 2 | 3 | 4 |
| --- | --- | --- | --- | --- | --- | --- |
|  | The **patient** asks questions or points out aspects he had not fully understood during the discussion. | 0 | 1 | 2 | 3 | 4 |
|  | **Clinician and patient** make sure that the patient can ask questions and point out aspects he had not fully understood during the discussion. | 0 | 1 | 2 | 3 | 4 |

| **12** | The **clinician** asks questions or points out aspects he had not fully understood during the discussion. | 0 | 1 | 2 | 3 | 4 |
| --- | --- | --- | --- | --- | --- | --- |
|  | The **patient** explicitly offers the clinician opportunities to ask questions or to point out aspects he had not fully understood during the discussion. | 0 | 1 | 2 | 3 | 4 |
|  | **Clinician and patient** make sure that the clinician can ask questions and point out aspects he had not fully understood during the discussion. | 0 | 1 | 2 | 3 | 4 |

| **13** | The **clinician** supports the patient in his activation of decision-making strategies. | 0 | 1 | 2 | 3 | 4 |
| --- | --- | --- | --- | --- | --- | --- |
|  | The **patient** talks about his decision-making strategies. | 0 | 1 | 2 | 3 | 4 |
|  | **Clinician and patient** discuss strategies for handling the decision. | 0 | 1 | 2 | 3 | 4 |

| **14** | The **clinician** opens the decision stage leading to the selection of an option (*If applicable, deferment is a possible decision*). | 0 | 1 | 2 | 3 | 4 |
| --- | --- | --- | --- | --- | --- | --- |
|  | The **patient** opens the decision stage leading to the selection of an option (*If applicable, deferment is a possible decision*). | 0 | 1 | 2 | 3 | 4 |
|  | **Clinician and patient** open the decision stage leading to the selection of an option (*If applicable, deferment is a possible decision*). | 0 | 1 | 2 | 3 | 4 |

| **15** | The **clinician** makes arrangements with the patient concerning how to proceed (*e.g. steps for implementing the decision, review of decision or of deferment*). | 0 | 1 | 2 | 3 | 4 |
| --- | --- | --- | --- | --- | --- | --- |
|  | The **patient** contributes towards the arrangements for how to proceed (*e.g. steps for implementing the decision, review of decision or of deferment*). | 0 | 1 | 2 | 3 | 4 |
|  | **Clinician and patient** discuss plans for how to proceed (*e.g. steps for implementing the decision, review of decision or of deferment*). | 0 | 1 | 2 | 3 | 4 |
