# Supplementary material for: MAPPIN'SDM – The Multifocal Approach to Sharing in Shared Decision Making
Source: PLoS One. 2012 Apr 13;7(4):e34849. doi: 10.1371/journal.pone.0034849 (PMC3325952; doi:10.1371/journal.pone.0034849)
Supplement: Appendix S2 — MAPPIN'SDM (doctor-) questionnaire. The MAPPIN'SDM questionnaire is supposed to be used by doctors assessing the communication quality in terms of SDM. The questionnaire comprises the same set of SDM indicators as the three foci of the MAPPIN'SDM observer instrument and the MAPPIN'SDM (patient-) questionnaire. In contrary to the observer instrument, scores have to be given based on subjective perception of the communication result rather than on behavioural attempts. The questionnaire was developed in German language and is provided here as (based on retranslation) investigator authorized English language version. (DOC) [file pone.0034849.s002.doc]

| **MAPPIN’SDM - Doctor and patient questionnaire for assessing consultations** | | | | | |
| --- | --- | --- | --- | --- | --- |
| Dear Doctor,  This questionnaire addresses both parties of a doctor-patient consultation about a medical decision (e.g. concerning treatment or examination). The aim is to ascertain to what extent both parties perceive the consultation in a similar way. It is thus important that both parties answer all questions independently of each other and directly after the consultation. | | | | | |
| **Please read all the questions before the consultation begins!** | | | | | |
| Apart from the reference, the questions are identical for doctor and patient, e.g.:  *“The patient understood the information…”* or accordingly:  *“I understood the information …”* | | | | | |
| Please mark the score ( ) that you consider most applicable. |  | | | | |
|  |  |  |  |  |  |
|  | **not at all** | |  | **absolutely true** | |
|  | 0 | 1 | 2 | 3 | 4 |
| **Your answers will not be shown to the other party!!** | | | | | |
| **Before you separate please agree on which decision(s) you talked about and fill in together which decision you will think about when filling in the questionnaire:**  (Please select only one) | | | | | |
| Please enter here: e.g. “Whether to start immunotherapy” or “Which prenatal tests” | (z. B.“Frage ob Aufnahme einer Immuntherapie“  oder „welche vorgeburtlichen Untersuchungen“) | | | | |
| **Important**: You should both be referring to the same decision. | | | | | |
|  |  | ***Thank you for completing this questionnaire!*** | | | |

| **1a** It was discussed which concrete medical problem requires a decision-making process. | **not at all** | |  | **absolutely true** | |
| --- | --- | --- | --- | --- | --- |
|  | **0** | **1** | **2** | **3** | **4** |
| **1b** The medical problem that requires a decision-making process is clear to the patient. | **not at all** | |  | **absolutely true** | |
|  | **0** | **1** | **2** | **3** | **4** |

| **2a** It was stated within the consultation that from a medical point of view there is not only one correct way to deal with my problem and that I cannot decide on my own which option is the right one in this case. | **not at all** | |  | **absolutely true** | |
| --- | --- | --- | --- | --- | --- |
|  | **0** | **1** | **2** | **3** | **4** |
| **2b** I am convinced that from a medical point of view there is not only one correct way to deal with this problem. Several basically equivalent ways are conceivable. The patient first has to clarify which of the respective pros and cons are important for him personally. It’s not possible for me as the doctor to know which option is the right one in this case. | **not at all** | |  | **absolutely true** | |
|  | **0** | **1** | **2** | **3** | **4** |

| **3a** It was discussed which approach should be used to exchange information within the consultation (*e.g. setting, verbal or graphic information*). | **not at all** | |  | **absolutely true** | |
| --- | --- | --- | --- | --- | --- |
|  | **0** | **1** | **2** | **3** | **4** |
| **3b** The way I exchanged information with the patient during the consultation suited both parties and contributed towards a mutual understanding (*e.g. setting, verbal or graphic information*). | **not at all** | |  | **absolutely true** | |
|  | **0** | **1** | **2** | **3** | **4** |

| **4a** It was discussed how the roles should be distributed during the consultation (*meaning: ‘balance of power’, distribution of responsibilities in the decision-making process*). | **not at all** | |  | **absolutely true** | |
| --- | --- | --- | --- | --- | --- |
|  | **0** | **1** | **2** | **3** | **4** |
| **4b** Role distribution during the consultation matched the patient’s wishes (*meaning: ‘balance of power’, distribution of responsibilities in the decision-making process*). | **not at all** | |  | **absolutely true** | |
|  | **0** | **1** | **2** | **3** | **4** |

| **5a** All the options were listed that are available for dealing with the current problem (*If applicable including that of doing without examination or treatment*). | **not at all** | |  | **absolutely true** | |
| --- | --- | --- | --- | --- | --- |
|  | **0** | **1** | **2** | **3** | **4** |
| **5b** The patient is aware of all the options for dealing with his current problem (*If applicable including that of doing without examination or treatment*). | **not at all** | |  | **absolutely true** | |
|  | **0** | **1** | **2** | **3** | **4** |

| **6a** The pros and cons of the different decision options were weighed up (*if applicable, also the pros and cons of the option to do without an examination and treatment*). | **not at all** | |  | **absolutely true** | |
| --- | --- | --- | --- | --- | --- |
|  | **0** | **1** | **2** | **3** | **4** |
| **6b** The patient now knows the pros and cons of the different decision options (*if applicable, also the pros and cons of the option to do without an examination and treatment*). | **not at all** | |  | **absolutely true** | |
|  | **0** | **1** | **2** | **3** | **4** |
| **7a** The patient’s personal expectations and fears about how to manage the concrete problem were discussed. | **not at all** | |  | **absolutely true** | |
|  | **0** | **1** | **2** | **3** | **4** |
| **7b** The patient’s personal expectations and fears went into the decision. | **not at all** | |  | **absolutely true** | |
|  | **0** | **1** | **2** | **3** | **4** |

| **8a** It was clarified what the medical information and recommendations are based on (*scientific evidence, my own judgement, benefits that I myself have when a certain measure is chosen, e.g. commission / research interests*). | **not at all** | |  | **absolutely true** | |
| --- | --- | --- | --- | --- | --- |
|  | **0** | **1** | **2** | **3** | **4** |
| **8b** It became clear to the patient what my medical information and recommendations are based on (*scientific evidence, my own judgement, benefits that I myself have when a certain measure is chosen, e.g. commission / research interests*). | **not at all** | |  | **absolutely true** | |
|  | **0** | **1** | **2** | **3** | **4** |

| **9a** It was checked whether the patient understood the information that I gave him. | **not at all** | |  | **absolutely true** | |
| --- | --- | --- | --- | --- | --- |
|  | **0** | **1** | **2** | **3** | **4** |
| **9b** The patient has understood the information that I gave him. | **not at all** | |  | **absolutely true** | |
|  | **0** | **1** | **2** | **3** | **4** |

| **10a** It was checked whether I have understood the patient’s viewpoint. | **not at all** | |  | **absolutely true** | |
| --- | --- | --- | --- | --- | --- |
|  | **0** | **1** | **2** | **3** | **4** |
| **10b** I understood the patient’s viewpoint. | **not at all** | |  | **absolutely true** | |
|  | **0** | **1** | **2** | **3** | **4** |

| **11a** Opportunity was provided to the patient to clear up the questions and aspects he / she had not fully understood during the discussion. | **not at all** | |  | **absolutely true** | |
| --- | --- | --- | --- | --- | --- |
|  | **0** | **1** | **2** | **3** | **4** |
| **11b** The patient cleared up the questions and aspects he had not fully understood during the discussion. | **not at all** | |  | **absolutely true** | |
|  | **0** | **1** | **2** | **3** | **4** |

| **12a** Opportunity was provided to me to clear up the questions and aspects I had not fully understood during the discussion. | **not at all** | |  | **absolutely true** | |
| --- | --- | --- | --- | --- | --- |
|  | **0** | **1** | **2** | **3** | **4** |
| **12b** I cleared up the questions and aspects I had not fully understood during the discussion. | **not at all** | |  | **absolutely true** | |
|  | **0** | **1** | **2** | **3** | **4** |

| **13a** Strategies to handle the decision were discussed (*i.e. how I will proceed when I make the decision*). | **not at all** | |  | **absolutely true** | |
| --- | --- | --- | --- | --- | --- |
|  | **0** | **1** | **2** | **3** | **4** |
| **13b** It has become clear to the patient what his decision-making strategy will be  (i. e. how the patient will proceed when he makes the decision). | **not at all** | |  | **absolutely true** | |
|  | **0** | **1** | **2** | **3** | **4** |

| **14a** During the consultation the decision stage was opened leading to the selection of an option (*If appropriate, the decision could be ‘to defer’*). | **not at all** | |  | **absolutely true** | |
| --- | --- | --- | --- | --- | --- |
|  | **0** | **1** | **2** | **3** | **4** |
| **14b** At the end of the consultation it was clear to the patient why and which decision was taken (*If appropriate, the decision could be ‘to defer’*). | **not at all** | |  | **absolutely true** | |
|  | **0** | **1** | **2** | **3** | **4** |

| **15a** It was discussed how to further proceed (*e.g. who has to inform whom; when the two of us will review the decision or the deferment*). | **not at all** | |  | **absolutely true** | |
| --- | --- | --- | --- | --- | --- |
|  | **0** | **1** | **2** | **3** | **4** |
| **15b** It is now clear to the patient how his problem will in future be dealt with (*e.g. who has to inform whom; when the two of us will review the decision or the deferment*). | **not at all** | |  | **absolutely true** | |
|  | **0** | **1** | **2** | **3** | **4** |
