# Supplementary material for: MAPPIN'SDM – The Multifocal Approach to Sharing in Shared Decision Making
Source: PLoS One. 2012 Apr 13;7(4):e34849. doi: 10.1371/journal.pone.0034849 (PMC3325952; doi:10.1371/journal.pone.0034849)
Supplement: Appendix S3 — MAPPIN'SDM (patient-) questionnaire. The MAPPIN'SDM questionnaire is supposed to be used by patients assessing the communication quality in terms of SDM. The questionnaire comprises the same set of SDM indicators as the three foci of the MAPPIN'SDM observer instrument and the MAPPIN'SDM (doctor-) questionnaire. In contrary to the observer instrument, scores have to be given based on subjective perception of the communication result rather than on behavioural attempts. The questionnaire was developed in German language and is provided here as (based on retranslation) investigator authorized English language version. (DOC) [file pone.0034849.s003.doc]

| **MAPPIN’SDM - Doctor and patient questionnaire for assessing consultations** | | | | | | | | | |
| --- | --- | --- | --- | --- | --- | --- | --- | --- | --- |
| Dear Patient,  This questionnaire addresses both parties of a doctor-patient consultation about a medical decision (e.g. concerning treatment or examination). The aim is to ascertain to what extent both parties perceive the consultation in a similar way. It is thus important that both parties answer all questions independently of each other and directly after the consultation. | | | | | | | | | |
| **Please read all the questions before the consultation begins!** | | | | | | | | | |
| Apart from the reference, the questions are identical for doctor and patient, e.g.  *“The patient understood the information…”* or correspondingly:  *“I understood the information… ”* | | | | | | | | | |
| Please mark the score ( )  that you consider most applicable. | |  | | | | | | | |
|  |  |  |  |  |  |  |  |  |  |
|  |  | **not at all** | | |  | | | **absolutely true** | |
|  |  | 0 | | 1 | | 2 | 3 | | 4 |
| **Your answers will not be shown to the other party!!** | | | | | | | | | |
| **Before you separate please agree on which decision(s) you talked about and fill in together which decision you will think about when filling in the questionnaire:**  (Please select only one) | | | | | | | | | |
| Please enter here: e.g. “Whether to start immunotherapy” or “Which prenatal tests” | |  | | | | | | | |
| **Important**: You should both be referring to the same decision. | | | | | | | | | |
|  |  | | ***Thank you for completing this questionnaire!*** | | | | | | |

| **1a** It was discussed which concrete medical problem requires a decision-making process. | **not at all** | |  | **absolutely true** | |
| --- | --- | --- | --- | --- | --- |
|  | **0** | **1** | **2** | **3** | **4** |
| **1b** The concrete medical problem that requires a decision-making process is clear to me. | **not at all** | |  | **absolutely true** | |
|  | **0** | **1** | **2** | **3** | **4** |

| **2a** It was stated within the consultation that from a medical point of view there is not only one correct way to deal with my problem and the doctor cannot decide on his own which option is the right one in my case. | **not at all** | |  | **absolutely true** | |
| --- | --- | --- | --- | --- | --- |
|  | **0** | **1** | **2** | **3** | **4** |
| **2b** I am convinced that from a medical point of view there is not only one correct way to deal with my problem. Several basically equivalent ways are conceivable. As a patient I first have to clarify which of the respective pros and cons are important for me personally. The doctor cannot decide on his own which option is the right one in my case. | **not at all** | |  | **absolutely true** | |
|  | **0** | **1** | **2** | **3** | **4** |

| **3a** It was discussed which approach should be used to exchange information within the consultation (*e.g. setting, verbal or graphic information*). | **not at all** | |  | **absolutely true** | |
| --- | --- | --- | --- | --- | --- |
|  | **0** | **1** | **2** | **3** | **4** |
| **3b** The way I exchanged information with the doctor during the consultation suited both parties and contributed towards a mutual understanding (*e.g. setting, verbal or graphic information*). | **not at all** | |  | **absolutely true** | |
|  | **0** | **1** | **2** | **3** | **4** |

| **4a** It was discussed how the roles should be distributed during the consultation (*meaning: ‘balance of power’, distribution of responsibilities in the decision-making process*). | **not at all** | |  | **absolutely true** | |
| --- | --- | --- | --- | --- | --- |
|  | **0** | **1** | **2** | **3** | **4** |
| **4b** Role distribution during the consultation matched my wishes (*meaning: ‘balance of power’, distribution of responsibilities in the decision-making process*). | **not at all** | |  | **absolutely true** | |
|  | **0** | **1** | **2** | **3** | **4** |

| **5a** All the options were listed that are available for dealing with my current problem (*If applicable including that of doing without examination or treatment*). | **not at all** | |  | **absolutely true** | |
| --- | --- | --- | --- | --- | --- |
|  | **0** | **1** | **2** | **3** | **4** |
| **5b** I am aware of all the options for dealing with my current problem (*If applicable including that of doing without examination or treatment*). | **not at all** | |  | **absolutely true** | |
|  | **0** | **1** | **2** | **3** | **4** |

| **6a** The pros and cons of the different decision options were weighed up (*if applicable, also the pros and cons of the option to do without an examination and treatment*). | **not at all** | |  | **absolutely true** | |
| --- | --- | --- | --- | --- | --- |
|  | **0** | **1** | **2** | **3** | **4** |
| **6b** I now know the pros and cons of the different decision options (*if applicable, also the pros and cons of the option to do without an examination and treatment*). | **not at all** | |  | **absolutely true** | |
|  | **0** | **1** | **2** | **3** | **4** |
| **7a** My expectations and fears about how to manage the concrete problem were discussed. | **not at all** | |  | **absolutely true** | |
|  | **0** | **1** | **2** | **3** | **4** |
| **7b** My personal expectations and fears went into the decision. | **not at all** | |  | **absolutely true** | |
|  | **0** | **1** | **2** | **3** | **4** |

| **8a** It was clarified what the medical information and recommendations are based on (*scientific evidence, doctor’s judgement, benefits that the doctor has if a certain measure is chosen, e.g. commission, research interests*). | **not at all** | |  | **absolutely true** | |
| --- | --- | --- | --- | --- | --- |
|  | **0** | **1** | **2** | **3** | **4** |
| **8b** It became clear to me what the medical information and recommendations are based on (*scientific evidence, doctor’s judgement, benefits that the doctor has if a certain measure is chosen, e.g. commission, research interests*). | **not at all** | |  | **absolutely true** | |
|  | **0** | **1** | **2** | **3** | **4** |

| **9a** It was checked whether I have understood the information the doctor gave me. | **not at all** | |  | **absolutely true** | |
| --- | --- | --- | --- | --- | --- |
|  | **0** | **1** | **2** | **3** | **4** |
| **9b** I understood the information the doctor gave me. | **not at all** | |  | **absolutely true** | |
|  | **0** | **1** | **2** | **3** | **4** |

| **10a** It was checked whether the doctor has understood my viewpoint. | **not at all** | |  | **absolutely true** | |
| --- | --- | --- | --- | --- | --- |
|  | **0** | **1** | **2** | **3** | **4** |
| **10b** The doctor understood my viewpoint. | **not at all** | |  | **absolutely true** | |
|  | **0** | **1** | **2** | **3** | **4** |

| **11a** Opportunity was provided to me to clear up the questions and aspects I had not fully understood during the discussion. | **not at all** | |  | **absolutely true** | |
| --- | --- | --- | --- | --- | --- |
|  | **0** | **1** | **2** | **3** | **4** |
| **11b** I cleared up the questions and aspects I had not fully understood during the discussion. | **not at all** | |  | **absolutely true** | |
|  | **0** | **1** | **2** | **3** | **4** |

| **12a** Opportunity was provided to the doctor to clear up the questions and aspects he had not fully understood during the discussion. | **not at all** | |  | **absolutely true** | |
| --- | --- | --- | --- | --- | --- |
|  | **0** | **1** | **2** | **3** | **4** |
| **12b** The doctor cleared up the questions and aspects he had not fully understood during the discussion. | **not at all** | |  | **absolutely true** | |
|  | **0** | **1** | **2** | **3** | **4** |

| **13a** Strategies to handle the decision were discussed (*i.e. how I will proceed when I make the decision*). | **not at all** | |  | **absolutely true** | |
| --- | --- | --- | --- | --- | --- |
|  | **0** | **1** | **2** | **3** | **4** |
| **13b** My decision-making strategy has become clear to me (*i.e. how I will proceed when I make the decision*). | **not at all** | |  | **absolutely true** | |
|  | **0** | **1** | **2** | **3** | **4** |

| **14a** During the consultation the decision stage was opened leading to the selection of an option (*If appropriate, the decision could be ‘to defer’*). | **not at all** | |  | **absolutely true** | |
| --- | --- | --- | --- | --- | --- |
|  | **0** | **1** | **2** | **3** | **4** |
| **14b** At the end of the consultation it was clear to me why and which decision was taken (*If appropriate, the decision could be ‘to defer’*). | **not at all** | |  | **absolutely true** | |
|  | **0** | **1** | **2** | **3** | **4** |

| **15a** It was discussed how to further proceed (*e.g. who has to inform whom; when the two of us will review the decision or the deferment*). | **not at all** | |  | **absolutely true** | |
| --- | --- | --- | --- | --- | --- |
|  | **0** | **1** | **2** | **3** | **4** |
| **15b** It is now clear to me how my problem will in future be dealt with (*e.g. who has to inform whom; when the two of us will review the decision or the deferment*). | **not at all** | |  | **absolutely true** | |
|  | **0** | **1** | **2** | **3** | **4** |
