# Supplementary material for: MAPPIN'SDM – The Multifocal Approach to Sharing in Shared Decision Making
Source: PLoS One. 2012 Apr 13;7(4):e34849. doi: 10.1371/journal.pone.0034849 (PMC3325952; doi:10.1371/journal.pone.0034849)
Supplement: Appendix S4 — MAPPIN'SDM coder manual. The MAPPIN'SDM coder manual provides comprehensive information and guidance for raters applying the MAPPIN'SDM approach. In particular, the manual comprises 64 pages and includes the following chapters: “Introduction", “Stage of research on SDM measurement", The MAPPIN'SDM method", “Using the manual", “The rater training", “Description of the indicators", “References", “Attachments". The coder manual was developed in German language and is provided here as investigator authorized English language version. (DOC) [file pone.0034849.s004.doc]

| 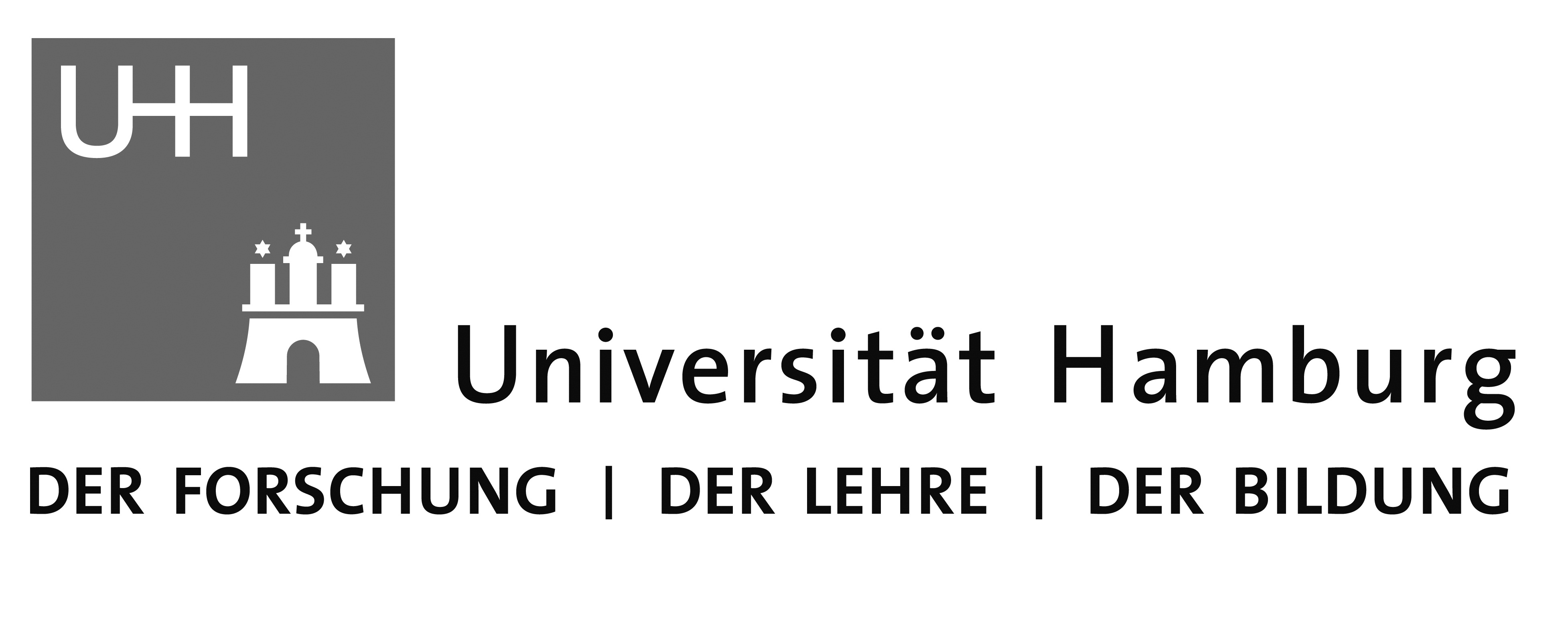 |  | |
| --- | --- | --- |
|  |  | |
| 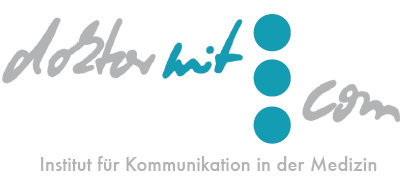 |  | |
| 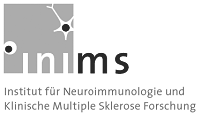 |  | |
| Manual for training and coding  MAPPIN’SDM  Multifocal approach to the **‘sharing’** in SDM | |  |
| **Jürgen Kasper** | |  |
|  | | |
|  | | |
|  | | |
|  | | |
|  | | |

**Version**:

July 2011

**Development**:

This manual was initiated and conceptualized in a student research project at the Unit of Health Sciences and Education with the participation of:

Frauke Hoffmann, Stefanie Matthies and Mirela Neukirchner

under the supervision of:

Jürgen Kasper.

It was carried out in the context of several funded research projects to develop and evaluate patient decision aids with the aim of enhancing the quality of

## communication between doctors and patients,

## Decisions made by these two parties, and

## The results of medical care.

Editorial and scientific review:

Frauke Hoffman, Katrin Liethmann, Friedemann Geiger

Translation and English language editorial office of the MAPPIN’SDM manual:

Yvonne Bulmer, Jürgen Kasper

Translation of the MAPPIN’SDM inventory (items):

Yvonne Bulmer

Re-translation:

Jutta Nickel

Consensus on final English version of the MAPPIN’SDM inventory:

Jutta Nickel, Yvonne Bulmer, Jürgen Kasper

**Contact:**

Jürgen Kasper, Dr. phil.

Institut für Kommunikation in der Medizin – Training und Forschung

Institute for Communication in Medicine – Training and Research

www. doktormit.com

k@sper.info

Unit of Health Sciences and Education

MIN-Faculty, University of Hamburg

Martin Luther King Platz 6

20146 Hamburg

Germany

and

Department of Primary Medical Care,

University Medical Center Hamburg

Martinistraße 52

Hamburg, Germany

[Introduction 4](#__RefHeading___Toc304362199)

[Stage of research on SDM measurement 5](#__RefHeading___Toc304362200)

[The MAPPIN’SDM method 6](#__RefHeading___Toc304362201)

[Using the manual 10](#__RefHeading___Toc304362202)

[The rater training 14](#__RefHeading___Toc304362203)

[Description of the indicators 17](#__RefHeading___Toc304362204)

[References 48](#__RefHeading___Toc304362205)

[Attachments 49](#__RefHeading___Toc304362206)

Introduction

This manual provides guidance on the usage of the MAPPIN’SDM observation instrument which has been developed to assess the mode and extent of patients’ involvement in their own medical decisions. It is to be applied to doctor-patient consultations about medical decisions. The observation instrument is part of a comprehensive inventory which enables the assessment of communication on decisions from all relevant perspectives (doctor, patient, observer). In addition to the observation instrument there are two questionnaires comprising identical items for addressing doctor and patient.

## The manual addresses participants of MAPPIN’SDM rater trainings and users of the observation instrument, for example in the context of communication analyses for research studies. Moreover, it addresses researchers who are seeking deeper insight into SDM (in particular MAPPIN’SDM) measurement methods and want to retrace the development of the inventory, and participants in communication training courses for medical decision-making. The manual was conceived to give all user groups sufficient and comprehensible information. It was a deliberate decision not to conceive separate documents as a way of solving the difficulty of varying interest profiles, (such as a short, easy to read version for use by physicians who take part in communication trainings). Instead, passages with more methodological content not essential to communication trainees were marked for easier orientation in the document. This decision is in line with the methodology of doktormit trainings which work on the basis of transparency, implying that information that is sought for is manageable whilst information that is not relevant to an individual will not be processed anyway. Hopefully, the chosen layout will help readers distinguish between the two kinds of material and encourage them to use the manual in accordance with their own individual interests.

Patient involvement is the core construct of the shared decision-making method – in contrast to the traditional style of paternalistic communication between doctor and patient. The traditional style of making decisions, where the physician acts like a father in taking on the patient’s responsibility is presumed to miss both parties’ needs and to impact negatively on the quality of the decision. In this context “quality” means that decisions are made by evidence informed patients in congruence with individual values.

‘Democratic’ communication is assumed to facilitate realization of a mutual exchange of medical knowledge, epistemic convictions, subjective values, previous experiences, and expectations. SDM requires the two competencies of mutual involvement in shared communication and consideration of the criteria of evidence based patient information (EBPI). The latter comprises international guidelines on how information about the benefits and drawbacks of medical measures should be given to patients to enable an unbiased understanding of the ‘risk information’. The criteria are based on either ethical guidelines or scientific evidence.

SDM enables patients with a pronounced desire for autonomy to shoulder a large part of the responsibility. Patients who lack confidence in their own critical assessment ability have the opportunity to ask for more guidance. What distinguishes this approach from the traditional paternalistic communication model is not so much the degree of patient involvement in the medical decisions but rather the extent to which the patient is involved in decisions on the very question of his involvement, i.e. in decisions on the communication process (meta communication). Observation using MAPPIN’SDM focuses on whether the players succeed in negotiating and clarifying role distribution explicitly and thereby providing good basic conditions for the information process. A consultation in which the doctor takes the responsibility can meet the SDM criteria if an agreement to this end has explicitly been made with the patient.

Stage of research on SDM measurement

Evaluation of SDM with regard to its effects on decision quality requires reliable methods for measuring SDM. The instruments that exist so far are not yet suitable for this purpose.

Systematic reviews on SDM measurement methods agree in their appraisal of existing instruments as unsatisfactory [Giersdorf 2004, Elwyn 2001, Simon 2007, Légaré 2007]. This appraisal of the international stage of research also applies to German research. As a result of the German ’patient as partner’ projects (BMGS 2001-2005) all the available Instruments for measuring SDM were translated into German. Problems of existing measurement approaches identified by the systematic reviews are listed below:

- Only few instruments address the quality of communication itself. Patient satisfaction / understanding / knowledge / preferences etc. do touch the SDM concept but they do not meet its core.
- The level of psychometric validation of some instruments is satisfactory as regards reliability but convincing validation studies are lacking [Simon 2007].
- None of the instruments tries to capture the interaction taking place between the parties involved. The subject of measurement is either the perception of the quality of communication or the relationship, or the completeness with regard to the sequence of process steps that should be fulfilled for an ideal decision. The issue of exchange (implying the extent to which the parties relate to each other) remains unconsidered, as does the question of whether there is a shared understanding of the central components and communicative aspects.

So far, studies have failed to recognize two further highly relevant problems of the used measurement methods:

- A lot of studies have revealed that the results of SDM measurements administered from different perspectives (third persons’ observations of SDM, subjective perception of patient or physician) do not, or only poorly, correlate [Saba 2006, Freiden 1980, Starfield 1981, Krones 2008, Kasper 2007, Shields 2005, Steward 2000, Martin 2003 Ogden 2002, Johnson 1988].

## Although claiming to provide information that is relevant to patients facing medical decisions, existing SDM measurements have so far not considered the quality of the information, thereby creating the absurd impression that patient involvement could take place without the patients being appropriately informed according to the guidelines of evidence based patient information.

Summing up, we can say that up to now, a definition and corresponding measurement methods that consider and integrate both the EBM criteria and the interactional character of the communication concept have been lacking. The latter implies the establishment of a shared understanding of the communicated meaning from different viewpoints, a `sharing of meaning` [Kasper 2011]

.

The MAPPIN’SDM method

Short overview of the MAPPIN’SDM approach

The development of MAPPIN’SDM was triggered by three aims:

**Firstly,** the MAPPIN’SDM observation instrument is in several respects a further development of the OPTION scale [Elwyn 2005] which it replaces with this more reliable, more comprehensive measurement method. However, this was not the most important goal of this development.

**Secondly,** MAPPIN’SDM is a research instrument that provides the opportunity to calculate convergent validities between assessments from different perspectives (doctor, patient, third person).

**Thirdly,** based on the inventory it is possible to calculate a compound SDM score that meets the concept’s assumptions (SDM_MASS_).

The structure of the MAPPIN SDM approach

## MAPPIN’SDM means: “Multifocal approach to the ‘sharing’ in the shared decision-making”. The inventory comprises all the relevant measurement perspectives towards the communication in question as well as the two possible measurement constructs (SDM behaviour, SDM result).

## These are applied to the three relevant measurement units (doctor, patient, dyad = doctor & patient as one unit). MAPPIN’SDM represents a comprehensive measurement approach integrating all perspectives, constructs and measurement units which were hitherto realized only separately in SDM measurement methods. This development answers the need for a systematic and exhaustive approach that captures and correlates all existing approaches.

Three perspectives

‘Perspective’ in this context means the viewpoint from which judgement of the communication is administered. In contrast to all existing instruments (which allocate SDM judgement either to the physician, the patient or a third person) MAPPIN’SDM realizes all three perspectives within the same inventory using an identical set of indicators.

Two constructs

**METHODS**

MAPPIN’SDM takes into account the fine but important difference between two constructs underpinning the existing SDM measurements (Construct in this context refers to the subject of measurement): **1. The extent to which a behaviour is performed to involve the two parties in the decision-making process: SDM Behaviour.** This issue can be judged not only by the patient and the doctor but also by a third person. The crucial question is: “Do doctor or patient undertake efforts to make the particular SDM issue explicit. *(and in doing so involve each other in the communication)?“* ***2.* The extent of involvement achieved: the perceived (communication) result in terms of SDM.** This issue can only be judged by parties immediately present and part of the consultation. The crucial question is: ”Did you feel involved in the communication on the particular issue during the consultation?“*.*  This means that, while the third person can only judge the communication (mediated) based on the first construct, parties immediately part of the communication can judge both constructs, for instance: behaviour: “The options were listed” or result: “Now I know my options”, which might not always be the same.

Three units of measurement

## Measurement unit refers to the (social) object upon which the measurements are made. In general, unilateral observation of communication cannot lead to valid judgements, in particular as the issue (involvement) is the product of an interpersonal cooperation. In addition to communication behaviour performed by the doctor as focused by the OPTION scale [Elwyn, 2003], MAPPIN’SDM takes into account contributions by the patient as well as the communication of doctor and patient as a unit (dyad). It is assumed that the quality of a decision does not depend solely on whether certain aspects are brought up, but also which of the parties brings up that specific aspect or to what extent both parties participate in the discussion of individual aspects. Full assessment of the process necessitates consideration of both dyad members.

Seven foci

**METHODS**

From these considerations result seven foci (see table). Focus is defined as the lowest common denominator of perspective, construct and unit (see table). The construct “behaviour” can be defined for three different perspectives (third person, doctor, patient) and also for three different units (doctor, patient, dyad), while the construct, ”result“, can be defined for two perspectives (not for third person) and two units (physician, patient). Strictly speaking, complete variation of the system would lead to another four foci which were not operationalized (instruments) since measurement of units and constructs by crossing over perspectives seemed rather complicated and was not required by the specific research questions. Foci 4 and 6 address ”behaviour“ as judged / perceived by doctor (4) and patient (6). Example: focus 6: “All options were listed”, focus 7: “Now I am aware of all the options available to me”. These measurement foci (4 and 6) are unusual since both patients and physicians can more easily respond to questions focusing on their perception of the result than on those reconstructing their observable behaviour. However, these two foci were operationalized to provide opportunity to interrelate a third person’s and subjective (patient’s or doctor’s) judgements based on identical constructs. Bearing in mind the strongly diverging results from third person and patient [Kasper 2007] it should be figured out, whether these could be due to differing constructs, which is not the case.

Two instruments

## These various foci of measurement are operationalized in two instruments, one of which is to be used as an observation based instrument (ideally using video documents), the other one to be administered (ideally directly after a consultation) by either patient or physician in the form of a questionnaire. This manual deals with the use of the observation instrument. However, the two questionnaires are attached at the end of the document.

Fifteen SDM indicators

All seven foci are based on an identical set of 15 aspects, covering an international consensus on competencies essential for SDM.

Since these competencies are captured as behaviours this does not mean that they would automatically lead to a sharing. Appearance of such behaviour just shows a person making efforts to achieve involvement. That is why we call these competencies ’indicators’. 11 indicators already exist in the OPTION scale [Elwyn 3003] and were adopted keeping their basic idea and, as far as possible, the wording. An authorized German translation already existed [Elwyn 2003] but, based on theoretic, language or communication considerations, some items had to be refined slightly so that they would fit better into the (MAPPIN’) SDM concept. These decisions are documented in detail in a comprehensive paper which records all the steps of the translation process [Kasper 2011]. In contrast to all existing instruments MAPPIN’SDM considers the criteria of evidence based patient information (EBPI [Bunge 2010]). For this purpose new indicators had to be defined. Moreover, the appraisal of existing indicators (OPTION) was specified according to EBPI criteria and explanations provided in further chapters of this manual. (e.g. Indicator 6). Readers already familiar with the OPTION scale can retrace changes below. (All indicators are commented on in detail in ”Description of the indicators”: p. 16 ff).

*Indicator 8* deals with the issue of indicating the source of any information or recommendation given. According to the EBPI criteria this information is standard [Bunge 2010]. Rather than being just a concept for organizing a dialogue the SDM concept is also concerned with information quality. Traceability of the information source is of particular relevance in the context of a face to face consultation since different kinds of information (scientific medical information, doctor’s beliefs, experiences or preferences) are naturally

provided in the same consultation. If no indication is made of the source for instance of a recommendation for a specific treatment option this information can be misleading. This issue is of relevance since it enables the exposure of potential conflicts of interests, e. g. when specific options are associated with any advantages on the doctor’s side.

**METHODS**

*Indicator 10* considers the question of whether the doctor has understood the patient’s viewpoint correctly. It corresponds to the already existing Indicator 9 (the question on the patient’s understanding). Since per definition SDM is a mutual exchange of information, reassurance about correct comprehension of the given explanations is required on both sides of the dyad.

*Indicator 12* corresponds to the already existing indicator that focuses on opportunities for the patient to ask questions. Accordingly, explicit communication indicating that the doctor be given opportunities to ask questions or express uncertainties would meet the idea of SDM.

*Indicator 13* defines an additional competency of meta-communication about decision-making strategies. It was defined based on observations made during analysis of comprehensive video material and was considered highly supportive, e.g. for involving the patient in negotiations on the preferred or previously experienced strategies for making important life decisions.

MAPPIN’SDM: overview of the system

| Focus | Perspective | Instrument | Construct | Unit |
| --- | --- | --- | --- | --- |
| 1 | observer | observation instrument | behaviour | doctor |
| 2 |  |  |  | patient |
| 3 |  |  |  | dyad |
| 4 | doctor | questionnaire |  |  |
| 5 |  |  | result |  |
|  |  | not operationalized | behaviour | patient |
|  |  |  | result |  |
| 6 | patient | questionnaire | behaviour | dyad |
| 7 |  |  | result |  |
|  |  | not operationalized | behaviour | doctor |
|  |  |  | result |  |

Quality of the observation based instrument

Ratings conducted by trained raters based on this manual have proven highly reliable. Both validation studies that were already conducted showed that although applying a system of pronounced complexity raters were able to achieve high inter-rater reliability (IRR). Results for IRR were comparable or even better than those reported for the OPTION scale [Elwyn 2003]. This might be due to the completion of the SDM indicators and communication foci. The introduction of additional categories facilitates the assignment of specific phenomena to the right category. Moreover, the specification of definitions in the coder manual by discourse in an interdisciplinary team of developers might have contributed to higher reliability. The manual includes clear guidance for differentiating all scoring levels, using possible double codings, and considering the relevance of the order of indicators in the sequence of the SDM indicators within a decision-making process. Encouraging experiences during the work with this instrument support the assumption of exhaustivity of the 15 SDM indicators.

Using the manual

Structure of the observer guide

1. The three variations of each indicator addressing the three measurement units (doctor, patient, dyad) are shown in a table using different backgrounds (dark grey = focus_doctor_, medium grey = focus_patient_, light grey = focus_dyad_). The indicators are the observation items.
2. Below each item is a definition of the indicator. Explanation is provided to illustrate the relevance of the indicator in the process of shared decision-making. The explanation also provides details about different sub-components of the indicator, distinction from other indicators, and varying degrees of performance.
3. In the following table the indicator is illustrated using concrete communication examples for each level of performance. Most of the examples are taken from our pool of consultations about multiple sclerosis treatment decisions. However, some quotations refer to other medical problems, e.g. consultation with the dentist or the GP.
4. Comments regarding the relevance of timing a behaviour in accordance with the course of the consultation and of possible double coding are in bold italics.

The meaning of the scores

| 0 | The behaviour is not observed. |
| --- | --- |
| 1 | The behaviour is observed as a minimal attempt |
| 2 | The basic competency is observed. |
| 3 | The behaviour is observed to a good standard. |
| 4 | The behaviour is observed to an excellent standard |

*Score »0« means:* No attempt to communicate the particular issue is observed at the right moment within the course of the consultation.

*Score »1« means:* The indicator is observed as a minimal attempt. This can for instance be an incomplete or rather implicit question or statement.

*Score* »2« *means*: The communication of the specific aspect is observed as a basic competency.

*Score »3« means:* Communication of the specific aspect is observed to a good or high standard. Explicit statements with additional explanations and/or descriptions referring to the issue in question are of a good standard.

*Score »4« means:* Comprehensive and remarkably insightful communication of the specific aspect is observed. Explicit statements with additional and extensive but also useful explanations and or descriptions referring to the issue in question should be coded as excellent performance.

How to code the dyad

## The idea of cooperation and partnership means that the process is open with regard to which of the parties initiates and performs specific decision-making steps. The traditional focus always looks at whether the doctor displays a particular behaviour. However, it might mean even more involvement if the patient initiates this behaviour himself. All things considered, it is crucial whether one of them takes steps to promote the decision-making process. The latter is the focus of the dyad, which should be either equal to or better than the best of the two scores of patient and doctor. A better score can be given when the parties’ contributions at least partly complement each other rather than being redundant, e.g. the patient starts listing the options and the doctor continues by adding an option the patient hasn’t yet listed. A minimal attempt to engage in one of the 15 behaviours is not enough to upgrade the performance of the other party substantially and thereby lead to a higher score for the dyad.

General coding rules

**METHODS**

The sole subject of the appraisal is decision processes, for instance a therapeutic or diagnostic question. A number of medical decisions can be made within a consultation or perhaps none at all. As a first step the decision sequence of reference has to be agreed on by defining accurate time marks on the video document. One possible procedure is that the decision that is considered most relevant in a consultation can be identified and then appraised. It is also possible to judge more than one process and to weight the SDM score in proportion to the consultation time of the specific sequence. Other methods for weighting SDM performance in the case of multiple decisions are also conceivable. However, it is recommended to prohibit appraisal of different decisions as one process, or to carry out appraisal of consultations that are without even one medical decision. If Indicator 1 is inappropriate or absent, this can make it difficult to recognize or to agree on the moment of initiation of a decision-making process. Researchers then have to try to infer the timepoint on the basis of the course and content of the consultation, e. g. in the case of a treatment decision by identifying the end of the medical history. It can happen that decision processes are more or less completely neglected, e. g. when the doctor initiates a diagnostic measure or decides on it without involving the patient in an information process. The raters should code such decisions indicator by indicator under consideration of this obvious lack of communication competency.

Responses (reactions) of one party to an action of the other party have to be considered by scoring the responding party only if the responder’s action goes beyond the response impelled by the initiator. If, for example, the doctor initiates Indicator 3 by asking the patient: ”Is this way of providing the information OK for you, or shall we switch to another medium?” and the patient just answers “It’s OK”, it is difficult for a rater to imagine less of an answer. The patient score is thus “0“. If, on the other hand, the patient answers by stating: ”I prefer it this way. If we do it a different way I will not be able to remember it“, this would be considered as patient activity for Indicator 3 leading to scores on both sides of the dyad.

Rarely observed indicators

According to our own and others’ experience some of the indicators that are included in the MAPPIN’SDM inventory are seldom observed.

This applies in particular to:

*Indicator 3:* - coming to an agreement on the preferred mode of information exchange

*Indicator 9:* - checking the patient’s understanding of information provided to him

*Indicator 10:* - checking the doctor’s understanding of information the patient contributed to the consultation

*Indicator 13:* - negotiating and counselling possible decision-making strategies

Recognition of these indicators is a challenge to the rater. They thus require special consideration within a rater training. Seldom events can easily be overlooked when they come as a surprise. (Apart from that, low or absent variance of a category leads to underestimation of actual rater agreement for statistical reasons [Wirtz & Caspar 2002]). There is naturally a reliability problem where seldom event categories are concerned. However, since the pool of indicators is defined on the basis of theoretic rather than empiric assumptions the observer’s attention has to be trained to recognize these indicators anyway. As a prerequisite for providing the opportunity to choose a suitable information mode (Indicator 3) it is for instance desirable that doctors have different variants available for the provision of patient information (e.g. decision aids). This applies in particular to prototypical medical problems such as stroke prevention in GP practices. However, in reality most physicians have not yet reached this standard and are not able to offer an alternative mode for informing their patients. Nevertheless, the competency should be considered in trainings and coded even if only as a minimal attempt.

Possibility of double coding

**METHODS**

In certain conditions, communication behaviours can indicate more than one competency. In such cases the same behaviour can give reason to score on different indicators. The manual provides guidance for deciding on possible double coding.

This is for instance possible for:

*Indicators 4 & 7:*- clarification of role distribution

- exploration of expectations and concerns

*Indicators 7 & 14:* - exploration of expectations and worries and

- initiation of the selection of an option

## Indicator 8 & 11: - discussing the source of a recommendation and

- opportunity to ask questions

## Double coding can also be justified in other than the aforementioned constellations. However, the opportunity of double coding should be used sparingly and not instead of a clear coding decision by the observer.

Coding depending on consultation course

The actual course of the decision-making process changes the basis of reference for appraisal of some of the 15 indicators.

This applies for instance to:

*Indicator 5 (listing the options):* Depending on how many options are available in a certain case the idea of what it would mean to list these options in a complete and comprehensive manner might vary. In an extreme case, when just one treatment option is available, the doctor would have to state: ”Apart from the option to defer the decision there is only one possible treatment available in your case.“ If more options are available Indicator 5 would require additional structuring to help the patient organize his plan of options.

*Indicator 14 (initiating a decision):* Depending on whether in the previous course of the consultation a choice was already expressed or whether up to this moment the possible options had only been discussed Indicator 14 requires a different strategy. In the first case, a feedback by the doctor on his understanding of the patient’s preference (making the implicit agreement explicit) would be appropriate. If the process still seems open an initiation of the decision could mean giving a summary of the remaining options. Examples are provided in the manual.

Coding of meta communication

Some indicators refer to communication competencies by making certain aspects of communication itself the subject of the consultation (= meta-communication). It is in line with the idea of SDM that patients are involved not only in the content relevant for the medical decision but also in decisions regarding the process, for example the course of the consultation or its setting.

**METHODS**

This applies to:

*Indicator 3* - agree on the preferred model of information exchange

*Indicator 4* - clarification of role distribution

*Indicators 11 & 12* - opportunity to ask questions

*Indicator 14* - initiation of the selection of an option

For these indicators it should be recognized that an implicit attempt to address the issue that is the subject of a certain indicator should be coded with “1”. To code the basic competency »2«, the explicit naming of the issue is required. If there is additional negotiation or discussion of the issue code »3« and in special cases »4« should be given.

The rater training

Training material

**METHODS**

Selection of communication examples for training rater competencies should be made very carefully. The aim is to achieve as heterogeneous a spectrum of communicative competencies as possible to that the participants can become familiar with the characteristics of different communicative qualities and levels. Since the structure of medical decisions varies between indications and settings, similarity of training materials and the consultations that will later be analysed is important. Raters trained using a certain material might need supplementary training to be able to reliably judge consultations from other health care contexts that have other specific conditions. It could be, for instance, that parts of the information are given by another carer in the unit, or that for particular reasons decisions are not taken but only prepared during the consultation in question. It can then be difficult to recognize specific indicators or criteria or to judge them reliably. Supplementary training enables renewed agreement on criteria for scoring and defining the decision sequences. It is usually necessary for raters to obtain information on the evidence basis of scientific knowledge from experts in the relevant medical field, for example concerning what are the available options or what the option of doing nothing in the face of a certain medical indication would mean?

Target criterion

Provided the training material has been carefully selected and all trainees have EBM competency the sole aim of the training is to achieve satisfactory inter-rater agreement, the aspired levels and coefficients of which can be decided on the basis of reference literature [Wirtz & Caspar 2002]. We assume that an average reliability of .80 based on a test sample of sufficient size is an acceptable result. This value reflects the result of a consensual validation achieved by all participants in the training. The training is a social process that takes place in a group. In other words, excellent individual performance is not relevant since the definition of the target criterion is based on the whole group.

Required time for trainings

It is difficult to estimate the time required for successful completion of a MAPPIN-SDM rater training. For some readers it might be important to learn that in our experience, despite its much greater complexity, MAPPIN does not require more training time than OPTION. This is assumed to be due to the higher accuracy of the coder manual and the exhaustivity of the indicator set which minimize uncertainties in rater assessments and save time otherwise needed for cognitive search processes and discussion of ambiguous explanations in the manual. Under optimized conditions a training might be realizable within five working days but this time can vary widely depending on the group and the material.

Selection of rater trainees

## To enable good results participants in rater trainings should meet various preconditions. Professional experience of social perception competencies such as basic psychotherapeutic skills is an ideal qualification for MAPPIN’SDM observer trainings but there should at least be a pronounced interest and motivation to discuss communicative phenomena in a group persistently and intensively. Observer agreement can only be achieved if all group members are capable of an open discourse. In addition to good basic medical knowledge and familiarity with health care procedures raters need good methodical skills in evidence-based medicine. They should in particular be able to apply the criteria of evidence-based patient information (EBPI [Bunge 2010, Kasper 2010]. Here MAPPIN differs clearly from other instruments (e.g. OPTION) which leave the question of the quality of the information out of consideration. Since patient involvement is not exhausted by creating an atmosphere of partnership but also requires that the best available relevant knowledge is shared with the patient in an understandable way, raters also have to be capable of appraising the information. If EBM or EBPI competencies are possibly lacking this has to be taken into account during the training. An important trainee selection criterion for this **elaborate and ex**pensive training is thus not only their availability at the time of the actual training but also in the future for analysing communication documents.

**MEHODS**

Didactics

The aim and scope of this manual does not allow for the provision of details on the didactics of observation (rater) trainings.

Description of the indicators

- For reasons of brevity the masculine pronoun ‘he’ is used in the following explanations (as throughout the manual) but it should of course be understood as also including the feminine pronoun ‘she’.

## Each indicator is explained using concrete communication examples for all scoring levels. These are to be understood as cumulative, i.e. each new level builds up on the lower levels and accordingly assumes their competencies. In other words, only those skills that are additional to the aforementioned skills are named.

## A corresponding training video was developed and produced in German and English. It demonstrates excellent performance of each of the following 15 indicators applied to a decision on immunotherapy in multiple sclerosis. The video shows a neurologist talking to a patient. The consultation is role play based on a prepared script.

Indicator 1: Defining the problem

| The **clinician** draws attention to an identified problem as one that requires a decision-making process. |
| --- |
| The **patient** draws attention to his concrete problem as one that requires a decision-making process. |
| **Clinician and patient** agree on a concrete problem as one that requires a decision-making process. |

This first competency consists in skills for defining the subject of the decision at issue.

- It is important to make clear which concrete problem requires a decision making process. Example 1: *multiple sclerosis:* “an inflammatory process of neural destruction. The question of immunotherapy arises“. Example 2: *stroke risk:* “the risk of blood congestion and blockage in small vessels of the brain to vascular closure leading to rupture. The question of a prophylactic treatment to thin your blood arises “. As the examples show, it is not just the diagnosis that has to be clarified but also the underlying process that gives cause for deciding on an intervention.
- A good performance includes discussion of the individual situation with regard to urgency and indication of treatment in a general sense, e.g. *immunotherapy in MS:* ”The frequency of relapses you are currently experiencing might indicate pronounced inflammatory activity. In such cases the chance of benefiting from immunotherapy is higher“; e.g. *stroke prophylaxis* ”Due to your age you belong to a group of people with an enhanced risk of stroke. Beyond that, your high blood pressure and the fact that you have already had one stroke point to a notably higher stroke risk compared to the normal population.“
- On the other hand it has to become clear what the decision at issue is about (e.g. whether or which treatment; or whether additional diagnostics and which).
- It is also essential to define the purpose of a medical measure on which a decision is to be made. Example: *immunotherapy in MS* ”You cannot expect immunotherapy to make existing impairments disappear. Immunotherapy can possibly slow down or even stop the progression of such impairments.”
- Explanations on how and by which mechanisms a treatment could affect the condition at issue score best if they are linked to the individual course of the disease or condition of the patient. Example: *stroke prophylaxis* ”A stroke becomes less likely when we reduce blood viscosity. We should now discuss whether thinning of the blood in your case would be wise or too dangerous.”

***Depending on whether there were previous appointments between the two parties, as on the context and specific circumstances leading to the current consultation the decision process can be initiated in a variety of ways. Even if the individual components required for a good performance are displayed in alternation with parts of the anamnestic process and are thereby performed step by step by doctor and patient together this can be rated a high performance. However, it is important to express these components explicitly and to demonstrate them at the beginning of the consultation, in particular before listing the options (Indicator 5).***

| **0** | **The behaviour is not observed:**  The concrete problem requiring a decision is not defined or is defined too late.  e.g. Clinician: “I’ve had a look at your medical results and I think we ought to do something.“  e.g. Patient: “Do you think I should take drug A or drug B? What do I have to do?“ |
| --- | --- |
| **1** | **The behaviour is observed (minimal attempt):**  The problem requiring a decision is defined or the kind of measure is mentioned on the basis of which a decision has to be made  e.g. Clinician: “Our job today is to come to a decision on immunotherapy for you.“  e.g. Patient: “My condition has got worse and I’d like to come to a decision today on a long-term therapy for my MS.“ |
| **In the three following, three further composites are exemplified, each of them leading to additional scoring points regardless of the specific combination in which they are observed in a concrete consultation:**   - **Making reference to the individual disease course**  Naming the aim / purpose of the measure on which a decision has to be made  - **Describing the mechanism of the measure and/or character of the specific disease process** | |
| **2** | **The basic competency is observed:**  In addition to point 1, it is defined which medical measure is the subject of decision. Moreover, it is stated why a decision has to be made. This requires reference to the individual course of the disease or condition and to the purpose of the potential measure.  e.g. Clinician: “… Your medical results show a relapsing-remitting course and a fast progression… Immunotherapy can slow down deterioration of your condition.“  e.g. Patient: ”… I know that there are several different drugs available for my MS type.” |
| **3** | **The behaviour is observed (good standard):**  In addition to point 2, details are provided on the mechanism of the medical measure under consideration.  e.g.Clinician: “… Immunotherapy intervenes in the body’s own immune system. It has an anti-inflammatory effect. That could prevent further damage to your nervous system.”  e.g. Patient: ”… I know that these drugs are anti-inflammatory.” |
| **4** | **The behaviour is observed (excellent standard):**  In addition to point 3, a link is successfully made between the mechanism and the individual case to allow for better estimation of potential benefit.  e.g. Clinician: “… Your MRT shows a lot of inflammatory activity in your nervous system. So immunotherapy would make sense there.“  e.g. Patient: “… My neurologist told me that the degeneration process of the nerves in my MS is not inflammatory and so immunotherapy probably won’t help.” |

***Indikator 2:*** Equipoise statement

| The **clinician** states that there is more than one way to deal with the identified problem (‘equipoise’). |
| --- |
| The **patient** indicates that there is more than one way to deal with the concrete problem. |
| **Clinician and patient** discuss that there is more than one way to deal with the concrete problem. |

A precondition for democratic decision-making in accordance with SDM is a mutual understanding on the part of both parties of “equipoise”, a term implying that the clinician’s medical knowledge does not qualify him – in the place of the patient – to know which treatment is right. SDM is indicated when more than one option is conceivable or justifiable. In addition to evidence-based information on the pros and cons of a treatment, clarification is therefore needed regarding what the possible benefits and drawbacks actually mean to the patient. By coming back to this essential issue on several occasions during the consultation it is possible to render the indicator more clear and credible. In contrast to the basic idea of partnership, an invitation to the patient to participate that is not based on a consensus according to equipoise would not contribute to SDM. This could rather imply the doctor was engaging in “cosmetic” communication and simply asking the patient to comply with the course of the discussion. It can therefore be stated that an agreement regarding equipoise between the parties is both catalyst and ’conditio sine qua non’ of SDM.

- The basic competency is given if it is stated explicitly that from the health professional’s point of view there are several conceivable ways to deal with the concrete problem.
- If this condition is also introduced and explained as the basic justification of patient involvement a higher level of competency is visible. Deeper discussion and justification of the equipoise condition are rated more highly. Excellent behaviour, for example, might be as follows: e.g*. immunotherapy for MS:* “No doctor can tell you how your MS will develop. Perhaps you will have a large number of relapses in the coming years. Or perhaps you will have none for 15 years. And no one can say whether you will belong to the group that is helped by therapy. As a doctor I can only make statements on the likelihood of a positive effect for different groups. For example, that in a group of 100 patients XY therapy will prevent relapses in 12 of them. No one can say whether you will be one of the 12 or one of the 88. And no one else knows how important it is to you to have less relapses and what you would do to achieve that. That is why your assessment of the situation is required.”

***It is important to demonstrate this competency already early on in the consultation, in particular before listing the options (Indicator 5). However, renewed mention of it at a later point in the consultation can be justifiable and lead to a higher score.***

| **0** | **The behaviour is not observed:**  The key messages of equipoise are not communicated or are communicated too late.  e.g. Clinician: “I recommend that you … / Our institute knows what’s good for you.“  e.g. Patient: “I am here to learn from you what I have to do.“ |
| --- | --- |
| **1** | **The behaviour is observed (minimal attempt):**  The key messages of equipoise are implicitly made an issue during the consultation by the clinician’s pushing the decision onto the patient.  e.g. Clinician: “I can’t tell you which is the best way for you.“ Or: “There are several ways of dealing with the problem.”  e.g. Patient: “I alone know what is right for me.“ |
| **2** | **The basic competency is observed:**  Equipoise is explicitly made an issue during the consultation by the clinician’s pointing out that he cannot make a decision in the patient’s place even if he has at his disposal strong evidence that in any way supports one of the possible options.  e.g. Clinician: “There are several ways of dealing with the problem. Based on scientific evidence it is not possible to say whether or which immunotherapy is right for you.”  e.g. Patient: “I know that medical facts can’t decide for me.“ |
| **3** | **The behaviour is observed (good standard):**  In addition to point 2, it is emphasized that the patient’s individual preferences pertaining to the specific decision are essential for appraisal of scientific evidence.  e.g. Clinician: “It’s ultimately a question of what the medical evidence means to you personally.“  e.g. Patient: ”… I have to weigh up for myself if it’s worth that for me ….” |
| **4** | **The behaviour is observed (excellent standard):**  Compared to point 3, an important focus is placed on the equipoise condition within the consultation. This is done by communicating the issue several times and in a particularly comprehensible way.  e.g. Clinician: “No doctor can tell you how your MS will develop. Perhaps you will have a large number of relapses in the coming years. Or perhaps you will have none for 15 years. And no one can say whether you will belong to the group that is helped by therapy. As a doctor I can only make statements on the likelihood of a positive effect for different groups. For example, that in a group of 100 patients XY therapy will prevent relapses in 12 of them. No one can say whether you will be one of the 12 or one of the 88. And no one else knows how important it is to you to have less relapses and what you would do to achieve that. That is why your assessment of the situation is required.”  e.g. Patient: “I have to judge for myself whether the chance to have one or two relapses less is incentive enough to start a therapy. You have given me an idea of how big that chance is.” |

***Indicator 3:* Preferred communication approach**

| The **clinician** ascertains the patient’s preferred approach to exchanging information (*e.g. in which setting, with which media, which time frame*). |
| --- |
| The **patient** participates in deciding on the preferred approach to exchanging information (*e.g. in which setting, with which media, which time frame*). |
| **Clinician and patient** choose an approach to exchanging information (*e.g. in which setting, with which media, which time frame*). |

The approach to information exchange is the specific way in which the parties provide each other with information. This third indicator refers to communication on “how” to proceed in this exchange. It includes decisions on the setting, location and participants. A patient who is involved in decisions about “how” to inform each other is much more likely to participate in the negotiation of “what” is to be discussed, i.e. which medical issue. Information exchange can be approached in different ways. In addition to print media, e. g. leaflets, diagrams or graphs, digital media can also be used. Merely mentioning other possibilities is not enough. It is crucial that doctor and patient clarify which approach best suits the situation and is preferred by the patient. An attempt to vary the communication approach (e.g. in response to a patient showing difficulties in understanding an issue) to achieve better understanding is seen as a minimal attempt.

- The competency is evident if the communication itself is made a subject of the consultation, e.g. by asking, “Would you prefer me to use a picture to explain this?” (basic competency).

## More comprehensive or thorough discussion of the setting can be awarded higher scores. Example: “OK, how shall we proceed? Shall we discuss the decision today? Shall I give you information to take home with you and then we make a new appointment? You would then have some time to think it over. Your wife could come with you next time, too, to say what she thinks. What do you say?”

***It is important to agree on the setting and mode of information exchange early on in the consultation.***

| **0** | **The behaviour is not observed.**  The way information will be exchanged is not made a subject of communication.  e.g. Clinician: “I’ll tell you a bit about it.“  e.g. Patient: The patient neither questions the way in which information is exchanged, nor does he make any other suggestions. |
| --- | --- |
| **1** | **The behaviour is observed (minimal attempt):**  Attention is paid implicitly to the issue of the approach to information exchange, e.g. by changing the medium in response to comprehension difficulties.  e.g. Clinician: (*Patient shows signs of comprehension difficulties*) “I can draw you a diagram. That might make it clearer.“  e.g. Patient: “Can you draw a diagram of that?“ Or: “Can you repeat that slowly so that I can write it down?“ |
| **2** | **The basic competency is observed:**  Attention is paid explicitly to the issue of the approach to information exchange, e.g. by asking questions or making suggestions.  e.g. Clinician: “Do you want me to go through the brochure with you or should I first give you an outline of what it says …?“  e.g. Patient: “Do you have a video that explains how the different therapies work?“ |
| **3** | **The behaviour is observed (good standard):**  In addition to point 2, alternative approaches and the patient’s preferences are discussed.  e.g. Clinician: “… What’s the best way for you to understand such explanations? Are you a visual person? Do you need written information to take home with you?”  e.g. Patient: “… I’d say I understand things better if they are visual. But what I mainly need is time… so that I can digest the information myself.” |
| **4** | **The behaviour is observed (excellent standard):**  In addition to point 3, alternative approaches are weighed up under consideration of the patient’s attitude and preferences.  e.g. Clinician: The doctor corrects his approach after conferring with the patient. “So how shall we proceed? Shall I give you information to take home with you and then we make a new appointment? You would then have some time to think it over. Your wife could come with you next time, too, to say what she thinks. What do you say?”  e.g. Patient: The patient determines the approach to information exchange by making a suggestion: “For me it would be good if you would first tell me a bit about it and then I could have time to read over it all again before meeting up for another appointment where my wife could be present when the decision is taken.“ |

***Indicator 4:* Distribution of roles**

| The **clinician** elicits the patient’s preferred level of involvement in decision-making. |
| --- |
| The **patient** expresses his preferred level of involvement in decision-making. |
| **Clinician** **and patient** discuss role distribution during the consultation. |

This competency refers to the distribution of roles during communication on the decision. The objective is to explore and discuss the patient’s idea of his part in the process. This may vary. One patient might prefer to leave the decision to his doctor. Another will prefer a shared process or even an autonomous decision. Merely asking the patient to express his expectations would not be sufficient (e.g. by using empty phrases), nor would it suffice to simply state how the roles will be distributed.

- The basic competency can be an explicit question or suggestion for the role distribution, e.g.: ”It looks to me as if that’s your decision.“
- To get a higher score a discussion has to be observed that elucidates and discusses the motives underpinning a specific role preference, e.g.: ”It’s your body, it’s your disease and therefore your decision, … Why do you want me to tell you what to do?”

## A more comprehensive discussion, within which role distribution is negotiated and a model is agreed on is judged excellent; e.g. ”Right! I see you would like to pass this thing on to me. Nevertheless, I still want to ask you again: What would you need to make your decision on your own?”

***It is important to clarify the roles early on in the consultation, i.e. before the options are listed (Indicator 5). Role clarification often occurs as a minimal attempt together with Indicator 7 (exploration of point of view, expectations, preferences and worries). If, in addition to an open question or exploration (Indicator 7), an offer of a social role can be understood this utterance can be double coded.***

| **0** | **The behaviour is not observed.**  Discussion of role preferences occurs too late in the consultation. The roles are taken without any meta communication on this topic.  e.g. Clinician: The clinician makes no attempts to find out what role the patient would like to play.  e.g. Patient: The patient accepts the role assigned to him without questioning. |
| --- | --- |
| **1** | **The behaviour is observed (minimal attempt):**  Role preferences are addressed only on the surface. This can be for example by indirectly alluding to the patient’s preferences regarding his role in the consultation. This would include patient behaviour that implicitly indicates his autonomy.  e.g. Clinician: “How’s it going?“ *(Trial balloon)*  e.g. Patient: “I’m not sure yet. I need more information.” |
| **2** | **The basic competency is observed:**  The preferred role model is explicitly made a subject of in the consultation.  e.g. Clinician: “Would you prefer to take the decision yourself or shall I take it for you?“  e.g. Patient: “I’ll come to a decision this evening with my husband.“ |
| **3** | **The behaviour is observed (good standard):**  In addition to point 2, the patient’s motives behind a specific preference are disclosed.  e.g. Clinician: “It’s your body, it’s your disease and therefore your decision, … Why do you want me to tell you what to do?“  e.g. Patient: “… You see I want to take your opinion into consideration because after all it’s you who will have to support me during the therapy.” |
| **4** | **The behaviour is observed (excellent standard):**  In addition to point 3, there is deeper discussion of role distribution and signals made by the participants are picked up. Moreover, the doctor undertakes efforts to strengthen patient autonomy.  e.g. Clinician: “Right! I see you would like to pass this thing on to me. Nevertheless, I still want to ask you again: What would you need to make your decision on your own?”  e.g. Patient: “… I’d like some information from you. I wouldn’t want to decide without consulting you. But in the end the decision is my own responsibility.” |

***Indicator 5:* Listing the options**

| The **clinician** lists the options (*If ‘doing nothing / deferring the decision’ is possible, this option should be included in the list*). |
| --- |
| The **patient** lists the options (*If ‘doing nothing / deferring the decision’ is possible, this option should be included in the list*). |
| **Clinician and patient** list the options (*If ‘doing nothing / deferring the decision’ is possible, this option should be included in the list*). |

The systematic listing of all relevant options can substantially contribute towards comprehension during the consultation. This applies to options concerning decisions on diagnostic procedures or on treatments. It is not enough to merely mention the different options during the course of the consultation. On the contrary, it is crucial that the options are presented in some sort of organized structure at the beginning of the consultation so that they can be picked up again and explained in more depth later on in the process.

- If an attempt is made to provide a list, but the list remains incomplete, e.g. because the doctor interrupts the listing process in order to give further explanations about drugs mentioned in the list, the competency is rated as a minimal attempt.
- The listing is only complete and the basic competency achieved if the option to do nothing is mentioned. The latter may appear in varying guises: It may be that some medical treatment is absolutely necessary so that it might seem that doing nothing is not a conceivable option. In such cases, however, it is often possible for the treatment to be of a provisional nature or it may be possible to defer the decision. Only in rare cases is the option of “doing nothing” (either provisional treatment or deferment) to be seen as irresponsible, for example in cases of emergency where SDM is not indicated anyway. If there is a choice between several medical options but doing nothing is not possible, it is recommended to express the urgency to act already at the beginning of the consultation (Indicator 1) when the concrete medical problem is introduced. Then, listing the options without “doing nothing” is reasonable and can be rated higher than basic competency. On the other hand, it should be considered that in cases where patients are already using a treatment there are two different options of “doing nothing”: continuing treatment and interrupting the treatment.
- Using attributes to help focus the patient’s mind on the individual option is rated a particularly outstanding performance, e.g. if when listing the options the doctor lines up objects on the table (one for each option). The doctor can then always hold the appropriate object in his hand when providing detail on one of the options. This competency can also be indicated if the doctor slows down an overhasty consultation course by taking a step back and listing the available options systematically.

***For this indicator it is important to be aware of the fact that the competency can never be rated higher than 2 if “to do nothing” is not among the listed options. The options should be listed prior to the communication of risks (Indicator 6).***

| **0** | **The behaviour is not observed.**  The listing of the options is omitted or comes too late in the consultation. Doctor and patient initiate the discussion of pros and cons of the different options without listing the options beforehand. |
| --- | --- |
| **1** | **The behaviour is observed (minimal attempt):**  The list of options is either incomplete or poorly structured, e.g. the options are difficult to distinguish as separate possibilities because the listing is already mixed up with more detailed explanations.  e.g. Clinician: “The available options are drug A, drug B which can have some rather unpleasant side-effects, and… Incidentally, a recently published study revealed that …. “  e.g. Patient: “Let’s list the possibilities. I know I can take drug M and – but I don’t think I would ever want to take that, because …. .“ |
| **2** | **The basic competency is observed:**  The relevant options are listed completely in a well-structured way. It becomes clear that the list involves separate options *(e. g. by using: either/or syntax).*  e.g. Clinician: “You can choose between drug B, drug A, or drug C.“  e.g. Patient: “According to a brochure I read about MS treatments I can choose between drug B, drug A or drug C.“ |
| **3** | **The behaviour is observed (good standard):**  Beyond being clearly structured the list is enriched by attributes for the different options.  e.g. Clinician: “There are three drugs: A, B, and C. A and B are injected daily, C weekly. And then D – a tablet – would also be feasible in your case, as would E, a monthly infusion. And of course there’s the possibility to wait and see how things continue without therapy.“  e.g. Patient: “Well, from the information I’ve worked through so far I have four possibilities… (*continuing as above …*).“ |
| **4** | **The behaviour is observed (excellent standard):**  The list can be rated ”4“ if in addition to a clear structure it includes references and individual comments that seem useful for the patient.  e.g. Clinician: “… You already know B, but you can take it in another dosage…, This might be of interest to you: a tablet, which would mean you wouldn’t have to inject yourself …“ |

***Indicator 6:* Pros & Cons**

| The **clinician** explains to the patient the pros and cons of the different options (*if applicable, also the pros and cons of ‘doing nothing’*). |
| --- |
| The **patient** discusses the pros and cons of the different options (*if applicable also the pros and cons of ‘doing nothing’*). |
| **Clinician and patient** weigh up the pros and cons of the different options (*if applicable, also the pros and cons of ‘doing nothing’*). |

This indicator is at the core of risk communication in that it deals with the likelihoods of benefits or harm resulting from a particular measure. The information has to be comprehensible and – equally important – it must be presented in an objective, well-balanced manner. This can be judged using the criteria of evidence based patient information [Bunge 2010].

- Biased discussion of benefits and side-effects cannot be given a score above “2” even if it is detailed. “Well-balanced” refers to benefits and side-effects and also to the relation between the presented options.
- If all the options appear in juxtaposition on an equal footing a higher score can be given. It is not enough just to list advantages and disadvantages (e.g. risks and side-effects) of the options. It is crucial that they are thoroughly explained. If the doctor succeeds in putting these explanations into the context of the patient’s individual disease course this reflects a high competency.
- The more extensive the information the greater the need for a structured approach to the explanations. If options or information regarding benefits or harm resulting from an option are not stated until later on this limits the score accordingly.

## The fine art of risk communication is to be seen in the consideration of the criteria of evidence based patient information (EBPI). For example, benefits and side-effects should be expressed in absolute numbers related to the same clearly defined reference group; it is necessary to explicitly provide a statement on the complementary non-benefit or non-side-effect rate, and this should also be expressed as absolute numbers of for instance 100 or 1000. The corresponding information is also needed for the option of doing nothing. In the case of diagnostic tests information on predictive accuracy (e.g. positive + negative predictive value), as on harm and benefits is required. Moreover, it is also necessary to declare the detection rate achieved without using the test and the consequences of possible test results (subsequent diagnostics and relevant details).

***For this indicator it is important to be aware of the fact that the competency can never be rated higher than “2” if the pros and cons of “doing nothing” are not included in the explanations. To get a high score it is necessary to connect explanations of pros and cons directly with the options (Indicator 5). Well-balanced information also implies that the doctor withholds any recommendations until the point at which the risk information is given in full.***

| **0** | **The behaviour is not observed.**  Advantages and disadvantages of the options are not a subject of the consultation |
| --- | --- |
| **1** | **The behaviour is observed (minimal attempt):**  Advantages and disadvantages of the different options are mentioned incompletely and/or not systematically.  e.g. Clinician: The doctor only talks about the benefits or only about the risks, and/or fails to include the option to do nothing.  e.g. Patient: “I read that most of the drugs that come into question for me have strong side-effects – hair loss and the like …“ |
| **2** | **The basic competency is observed:**  Advantages and disadvantages of the different options are discussed systematically, i.e. they are assigned to the corresponding option. An idea is given of the probability of benefits or harm resulting from the individual options.  e.g. Clinician: “Drug C can lead to a reduction in the number of relapses. It works about as well as drugs A and B… The advantage of drug B is that it only has to be taken twice a week, whilst C has to be injected daily. The main side-effect is skin changes at the injection site. But that’s very rare …“  e.g. Patient: “Drug C can reduce the number of relapses. It works about as well as drugs A and B… The advantage is that I only have to take this drug twice a week whilst I have to inject C every day. The main side-effect is skin changes at the injection site. But that’s very rare …“ |
| **3** | **The behaviour is observed (good standard):**  In addition to point 2, (including the possibility of doing nothing) more detailed explanations are provided and support is given for appraising the pros and cons: e.g. the doctor explains the mechanism by which a drug acts in the disease process or the reasons for side-effects. Reference is made to the patient’s individual disease course.  e.g. Clinician: “Although it’s generally true that drug B is most likely to slow down disease progression you experienced a worsening of symptoms under that treatment. So, despite the fact that it’s not possible to say certainly that drug B has no effect at all on your disease it does rather seem to point in the direction of trying a different therapy.“  e.g. Patient: “Although it’s generally true that drug B is most likely to slow down disease progression I experienced a worsening of symptoms under that treatment. Now I know that doesn’t mean that drug B has no effect at all on my disease but does it perhaps suggest that I should try a different therapy?“ |
| **4** | **The behaviour is observed (excellent standard):**  Communication of pros and cons and the clarification of the likelihood of their occurring lead to a balanced picture. For example, a short summary is given at the end of a longer explanation. Another possibility is to adapt the structure of the explanations to the structure of the patient’s knowledge or expectations. It should also be judged as excellent performance if a good explanation is linked to the message of uncertainty: “It is not possible to know whether you would be in the group of patients who benefit.” Due to lack of space it is not possible to present all the EBPI criteria that would lead to a score of “4”. The criterion can be considered fulfilled if an EBPI-based decision aid is used.  e.g. Clinician: “… Right, those were the two options you are already familiar with. There is also a new therapy ….“  e.g. Patient: “… OK, those were the two options that I know already. There is also a new therapy ….“ |

***Indicator 7:* Expectations & worries**

| The **clinician** explores the patient’s expectations (*ideas*) and concerns (*fears*) about how to manage the concrete problem. |
| --- |
| The **patient** describes his expectations (*ideas*) and concerns (*fears*) about how to manage the concrete problem. |
| **Clinician and patient** discuss the patient’s expectations (*ideas*) and concerns (*fears*) about how to manage the concrete problem. |

This indicator refers to the communication of the patient’s expectations (ideas) and worries (anxieties) with regard to the management of the problem. What presuppositions does the patient have about what should be done? What are the patient’s preferences regarding for example the mode of application? What worries the patient? What underpins the patient’s point of view? Communication of these patient-related aspects is crucial in SDM.

- Of relevance to the rating of a minimal attempt or the basic competency is the question of to what extent the patient’s perspective becomes explicitly subject of the communication, e.g. *immunotherapy of MS*: ”Would you spontaneously prefer any of the options I explained to you? … Do my explanations meet your own ideas? … Now we have talked in detail about the procedures, chances of benefit, risks, side-effects and so on regarding a possible treatment with drug A. What do you think about it now?”
- Better performance on the doctor’s part is shown by expanding the single question (basic competency) to a more comprehensive exploration (including probing and comments on the patient’s attitude). On the part of the patient, carefully considered, well-founded statements regarding his own point of view are rated higher than mere expression of preferences and worries, e.g. *immunotherapy of MS* “… In addition to the concrete pros and cons of the treatment options, are there any significant aspects that will be of importance to you personally when you make your decision? How do you see yourself when it comes to managing a serious disease? “

***This indicator can be double coded with Indicator 4. Utterances of clear/ strong preferences in the sense of a decision or pre-decision can also be coded, if applicable through double coding with Indicator 14. If the doctor obviously ignores the patient’s preferences this negligence can be reflected by removing points given at an earlier stage in the consultation (for the same Indicator 7).***

| **0** | **The behaviour is not observed.**  No exploration of the patient’s expectations and worries can be observed.  e.g. Clinician: (*following the patient’s statement that “These flu-like side-effects worry me quite a bit when I imagine having them several times a week*…”): “I think you should start the therapy quite soon …“) |
| --- | --- |
| **1** | **The behaviour is observed (minimal attempt):**  The patient’s expectations and worries are explored superficially, e.g. by using an open question to ask the patient for an atmospheric picture, or (patient) by indicating a feeling of uncertainty but failing to clarify exactly what it is.  e.g. Clinician: “So, how about it?“ Or: ”What do you think?“  e.g. Patient: ”I don’t really know how I feel about Drug B“ |
| **2** | **The basic competency is observed:**  The patient’s expectations and/or worries are an explicit part of the consultation.  e.g. Clinician: “Do you have the feeling that any one of the treatment options I’ve just explained fits you particularly well? … Do the explanations I just gave you more or less meet your expectations? … We’ve talked in detail about the procedure, risks and side-effects of treatment with drug A. What do you think now about therapy with that drug?”  e.g. Patient: “… Now that you’ve told me that there can be a lot of side-effects during therapy with drug B I have to say I’m rather frightened. How can I manage all that with two children?” |
| **3** | **The behaviour is observed (good standard):**  In addition to point 2, more far-reaching questions/ statements by the patient on worries or ideas are discussed.  e.g. Clinician: “… I can understand your concern about the treatment’s side-effects but I can reassure you: studies have shown that these serious side-effects only occur in 5 out of 100 patients.”  e.g. Patient: “… I can’t afford to be ill in bed all the time. I’ve weighed up the possible advantages against the drawbacks of therapy and to be honest the bottom line is that I’ll do without therapy.” |
| **4** | **The behaviour is observed (excellent standard):**  In addition to point 3, these aspects are discussed in depth and/or reference is made to the individual decision-making process.  e.g. Clinician: ”In addition to the concrete pros and cons of the treatment options, are there any significant aspects that will be of importance to you personally when you make your decision? How do you see yourself when it comes to managing a serious disease? “  e.g. Patient: “… I see my MS as a task I have to handle. I’m not basically against taking a drug but as I see it, the discussion of this treatment – particularly since its effects are very uncertain – leads me away from what it’s really all about for me.“ |

***Indicator 8:* Indicating source of recommendation / information**

| The **clinician** makes known to the patient the source upon which his information / recommendations are based (*scientific evidence, own judgment, preferences, conflicting interests*). |
| --- |
| The **patient** clarifies the source upon which medical information / recommendations are based (*scientific evidence, clinician’s judgement, preferences, conflicting interests*). |
| **Clinician and patient** clarify the source upon which medical information / recommendations are based (*scientific evidence, clinician’s judgement, preferences, conflicting interests*). |

This indicator refers to the competency to declare the specific source of medical information and recommendations. It is one of the standards of evidence based medicine to state a reference for scientific proof. This standard also applies to the communication of medical data to patients in a face to face consultation or for example using a print information tool. This requirement might seem artificial to doctors in the context of risk communication during a consultation. However, it is even more important here than in other settings: The SDM concept intends that the opportunity for doctor and patient to express personal opinions is provided. The doctor’s opinion is very important to the patient. An informed choice is not, however, possible if the patient cannot differentiate between information that represents the doctor’s opinion and subjective experiences and information that is based on scientific evidence or, as another possibility, the guidelines of the specific hospital. Moreover, it is important to declare the doctor’s potential conflicts of interest, e.g. there might be a conflict between his personal observations and appraisal on the one hand and possible financial incentives from a pharma company on the other hand.

- Performance here stands and falls on the accuracy with which the background to any statements is explained; this means a superficial hint such as for example “Our head doesn’t like it” does not suffice. It is not enough just to refer to the source of any information.
- The communication that is crucial for this indicator is the particularized portrayal of the results drawn from a specific source as well as a critical appraisal of scientific quality and the possible usefulness of this information for the patient, e.g. *immunotherapy of MS*: ”In a large placebo controlled study 7 out of 100 patients using drug C had a drug related benefit in that they experienced less relapses.“ and ”This result might be relevant for you if you say: Reducing the frequency of relapses is an important goal for me.“ or ”This study seems to me to be of more relevance for you compared to other studies showing no benefit of drug C. The participating patients were more comparable with you with regard to their disease course etc.“.

| **0** | **The behaviour is not observed.**  No reference is made to the origin of a recommendation or information provided in the consultation.  e.g. Clinician: “We strongly recommend you to take drug M.“  e.g. Patient: The patient makes no effort to question the origin of the recommendation. |
| --- | --- |
| **1** | **The behaviour is observed (minimal attempt):**  The source of a recommendation/ information is mentioned superficially, e.g. using an empty phrase which does not help to establish the quality of the information.  e.g. Clinician: “Based on our institute’s experience …“, ”From a medical point of view …“ Or: “Viewed scientifically …“  e.g. Patient: “Is that based on your experience?“ |
| **2** | **The basic competency is observed:**  The source of the recommendation(s) / information is clearly indicated by referring to a certain background (e.g. either a personal preference or a scientific source).  e.g. Clinician: “I recommend you start a therapy with drug C since studies have shown an advantage over other immunotherapy drugs.“  e.g. Patient: “Are your recommendations based on scientific findings or on your experience as a doctor?“ |
| **3** | **The behaviour is observed (good standard):**  In addition to point 2, a conclusion drawn from or the relevance given to the particular source is explained.  e.g. Clinician: “In a big placebo controlled study 7 out of 100 patients experienced benefits from taking drug C since they had less relapses.“  e.g. Patient: “So you’re recommending I have therapy with drug A. But are there studies to back up your statement? Did participants have the same disease course as mine?” |
| **4** | **The behaviour is observed (excellent standard):**  Compared to point 3, the explanations are more exhaustive, e.g. by weighing the relevance of diverging information from different sources against each other.  e.g. Clinician: “The study seems more meaningful to your case than another one which revealed no benefit for drug C. The patients that took part were much more comparable with you.”  e.g. Patient: “How often and on which patients were these operations carried out already?“ |

***Indicator 9:* Evaluation of patient’s understanding**

| The **clinician** checks that the patient has understood the information. |
| --- |
| The **patient** clarifies how he understood the information given by the clinician. |
| **Clinician and patient** clarify whether the patient understood the information given by the clinician correctly. |

This indicator concerns the competency to check the patient’s understanding of the information provided by the doctor. Like the other SDM indicators, this skill can be initiated either by the patient or by the doctor. In addition to reassurance of comprehension it is important to probe for any lack of knowledge or uncertainties on the patient’s side.

- Neither a question by the doctor as to whether the patient has understood, nor brief confirmation by the patient, can be considered sufficient.
- On the contrary, how the patient understood the information should be made a subject of the consultation.
- A high level of competency is indicated if the patient’s understanding is checked by direct questioning or if the patient communicates his understanding by providing a well-structured representation of what he was told. This understanding should additionally be put into the context of the information provided by the doctor, e.g. “… How would you sum that up for yourself? Could you tell me what you have understood so far?“
- An excellent performance yields reassurance of the patient’s comprehension at each important stage of the consultation without, however, treating the patient in a schoolmasterly manner. It is assumed that excellent performance of this indicator can only be achieved cooperatively.

| **0** | **The behaviour is not observed:**  The patient’s understanding is not made a subject of the consultation.  e.g. Clinician: “Could you understand me?“  e.g. Patient: “I understood so far.” |
| --- | --- |
| **1** | **The behaviour is observed (minimal attempt):**  The patient’s understanding of the given information is checked fleetingly (e. g. using empty phrases).  e.g. Clinician: “How well did you understand that?“  e.g. Patient: ”So it gives you flu-like symptoms, does it?“ |
| **2** | **The basic competency is observed:**  Explicit statements and/or questions signify that the patient’s understanding of the given information is an issue.  e.g. Clinician: ”How well did you understand the different possible procedures?“  e.g. Patient: “If I understood you correctly, drug B causes flu-like symptoms.“ |
| **3** | **The behaviour is observed (good standard):**  In addition to point 2, particular emphasis is paid to specific aspects (by further questioning and/or by detailed reconstruction).  e.g. Clinician: “… How would you sum that up in your own words? Could you just tell me what you have grasped so far?“  e.g. Patient: “… And so compared to drug C it’s less dangerous. But the two don’t differ with regard to dosage. Is that right?“ |
| **4** | **The behaviour is observed (excellent standard):**  The performance is excellent if it succeeds in making sure of the patient’s understanding at each important stage of the consultation without treating the patient in a schoolmasterly manner. It is assumed that excellent performance of this indicator can only be achieved cooperatively.  e.g. Clinician: ”… Let’s sum that up and see what it means to you“  e.g. Patient: ”… What I understood is that compared to drug B, drug C is less risky but similarly efficient”  Clinician: ”That’s right, as far as we know it’s similarly efficient with regard to the reduction of relapse frequency”  Patient: “But maybe not regarding progression of handicap“  Clinician: “Right, that’s it” |

## Indicator 10: **Evaluation of doctor’s understanding**

| The **clinician** makes sure that he has understood the patient’s viewpoint correctly. |
| --- |
| The **patient** makes sure that the clinician understands his viewpoint. |
| **Clinician and patient** clarify whether the clinician has understood the patient’s viewpoint correctly. |

Accordingly (cf. Indicator 9) the parties should clarify the doctor’s understanding of the patient’s contributions. In addition to reassurance of comprehension it is important to probe for any lack of knowledge or uncertainties on the doctor’s side.

- Neither an incidental reassurance by the patient that the doctor understood him correctly nor brief confirmation by the doctor that he is able to follow the patient’s explanations can be considered sufficient.
- On the contrary, the doctor should rather be asked to reconstruct his understanding of the patient’s point of view or he could do this spontaneously. The level of communication is higher if the doctor’s reconstruction is close to the patient’s utterances.

| **0** | **The behaviour is not observed:**  The doctor’s understanding is not made a subject of the consultation.  e.g. Clinician: “I understand.“  e.g. Patient: “Could you follow me?“ |
| --- | --- |
| **1** | **The behaviour is observed (minimal attempt):**  The doctor’s understanding of the patient’s point of view is checked fleetingly (e.g. using empty phrases).  e.g. Clinician: “Oh, that’s it, is it?“  e.g. Patient: “Do you understand where my exact problem is – Do you know what I mean?” |
| **2** | **The basic competency is observed:**  Explicit statements and/or questions signify that the doctor’s understanding of the patient’s point of view is an issue.  e.g. Clinician: “Have I understood you correctly that you are of the opinion that you should have opted for immunotherapy much earlier on?“  e.g. Patient: “Just to make sure you understand me correctly: What I said was that I am not basically against drug therapy …“ |
| **3** | **The behaviour is observed (good standard):**  In addition to point 2, particular attention is paid to specific aspects (by further questioning and/or by detailed reconstruction).  e.g. Clinician: “You’ve led me to understand that you want to change something because….“  e.g. Patient: “What do you make of my explanations? I get the feeling you haven’t quite grasped what I mean!“ |
| **4** | **The behaviour is observed (excellent standard):**  In addition to point 3, individual aspects are put into context with the patient’s contributions, and there is an interactive discussion of the patient’s point of view.  e.g. Clinician: “From listening to you just now I understand two different aspects: Firstly, that you’re not basically against immunotherapy and that you recognise the possible benefit. On the other hand I get the impression you don’t want to make a decision today. There’s still something that’s bothering you. Is that what you’re saying?  e.g. Patient: “In part, yes, but I have to correct you on one point. I’ll explain it differently.“ |

## Indicator 11: **Opportunity for questions (from patient)**

| The clinician explicitly offers the patient opportunities to ask questions and to point out aspects he had not fully understood during the discussion. |
| --- |
| The clinician explicitly offers the patient opportunities to ask questions and to point out aspects he had not fully understood during the discussion. |
| Clinician and patient make sure that the patient can ask questions and point out aspects he had not fully understood during the discussion. |

The corresponding behaviour reflects the competency to provide sufficient opportunity for the patient to ask questions and express aspects that he has not yet fully understood. Such behaviour should be part of everyday communication but here the idea is to emphasize the aforementioned opportunity by explicitly inviting the patient to ask questions.

- This means that neither a friendly atmosphere which gives the opposite party the feeling that there is enough space for questions during the consultation nor the doctor’s willingness to answer any questions is sufficient for a basic competency.
- The basic competency is only achieved if there is an explicit invitation to ask questions or if the patient asks questions spontaneously.
- Higher levels of this competency are indicated if there is more frequent, more specific encouragement to ask questions of if, in addition, the doctor probes deeper to enable a better understanding of the patient’s questions and underlying concerns. On the part of the patient a higher level of this competency is indicated by a greater number of questions, as by the persistence and quality of the questions.

***Any patient questions can be coded which appear during the decision-making process (up to the point where the decision is made). Questions that are expressed after the decision may be considered under Indicator 15 which deals with the discussion of further procedure (Indicator 15). Questions referring to the patient’s worries and concerns should be coded under Indicator 7. If a question refers to both a patient’s worries and the information process, double coding (Indicators 7 and 11) is possible. Additionally, questions referring to Indicator 8, the source of information, can be double coded here.***

| **0** | **The behaviour is not observed:**  The patient does not ask any questions and does not seem to have the opportunity. |
| --- | --- |
| **1** | **The behaviour is observed (minimal attempt):**  The patient’s questions are stated implicitly or the opportunity to ask questions can be assumed from the atmosphere or the doctor’s reaction to questions.  e.g. Clinician: (e.g. by pauses in speech, visual contact)  e.g. Patient: (by utterance of a thoughtful sigh) |
| **2** | **The basic competency is observed:**  The patient explicitly asks questions and/or the doctor invites the patient to do so.  e.g. Clinician: “Any questions on this topic?“  e.g. Patient: “There’s one thing I don’t understand …“ Or: “Can I ask one more question?“ |
| **3** | **The behaviour is observed (good standard):**  In addition to point 2, any as yet unsolved questions on the part of the patient are picked up again, leading to more details and clarification.  e.g. Clinician: “What makes you ask this question? What is it that interests you here? Is it … or …?“  e.g. Patient: “I need to put my question more precisely: You just said … I haven’t understood that yet.“ |
| **4** | **The behaviour is observed (excellent standard):**  In addition to point 3, communication on questions expressed by the patient leads to the emergence of new, more specific or more far-reaching aspects. These can be stated by the doctor or the patient.  e.g. Clinician: “I’ve sometimes asked myself …“ “Is that a question that’s bothering you?“  e.g. Patient: “I’ve understood the effects and side-effects but I now ask myself whether the two are connected. What I mean is, if the drug doesn’t have the desired effect for me can I expect the probability of side-effects to be the same, (i.e. no effect = no side-effects)?“ |

***Indicator 12:* Opportunity for questions (from doctor)**

| The **clinician** asks questions or points out aspects he had not fully understood during the discussion. |
| --- |
| The **patient** explicitly offers the clinician opportunities to ask questions or to point out aspects he had not fully understood during the discussion. |
| **Clinician and patient** make sure that the clinician can ask questions and point out aspects he had not fully understood during the discussion. |

In the interests of reciprocity within the information process this indicator refers to the opportunity that the doctor is given to ask questions and express aspects that he has not fully understood. These might include uncertainties associated with the decision-making process or with the information provided by the patient. They should not, however, be about the diagnostic or medical history process within the consultation even if they are asked in its further course. The competency that is to be judged here can be shown either by the patient or by the doctor. Coding is as for Indicator 11.

***Doctor’s questions concerning the medical history are not coded here. This indicator must also be rated separately from the aspect of reassurance whether the information given was also understood (Indicator 9). Questions referring to worries and concerns as expressed by the patient can only be coded under Indicator 7.***

| **0** | **The behaviour is not observed:**  The doctor does not ask any questions, nor does he have the opportunity to do so. |
| --- | --- |
| **1** | **The behaviour is observed (minimal attempt):**  The doctor’s questions are implicit and/or the general atmosphere makes the opportunity for questions evident. On the part of the patient this competency can be observed for example as pauses during speech, or in his intonation, on the part of the doctor by his signifying a need to ask a question or be given more information.  e.g. Clinician: “I’m not familiar with the website you visited but to my knowledge …“  e.g. Patient: (*when reporting information gained in a chat forum*) ”… You probably know the website …“ |
| **2** | **The basic competency is observed:**  The doctor asks explicit questions and/or the patient explicitly encourages the doctor to do so.  e.g. Clinician: “To what extent does your desire to have children play a role in your decision on immunotherapy?“  e.g. Patient: ”Is there anything else you need to know here?“ |
| **3** | **The behaviour is observed (good standard):**  In addition to point 2, any as yet unsolved questions on the part of the doctor are picked up again, leading to more details and clarification.  e.g. Clinician: “Would you like to become pregnant in the near future if possible or is it a longer-term wish?“  e.g. Patient: “Would you like me to give you the internet link so that you can look up where I got my information? “ |
| **4** | **The behaviour is observed (excellent standard):**  In addition to point 3, communication on questions expressed by the doctor leads to the emergence of new, more specific or more far-reaching aspects. These can be stated by the doctor or the patient.  e.g. Clinician: “But there’s one thing I still haven’t understood…, because just now you said… Why the change of mind?“ |

## Indicator 13: **Supporting strategies of decision-making**

| The **clinician** supports the patient in his activation of decision-making strategies. |
| --- |
| The **patient** talks about his decision-making strategies. |
| **Clinician and patient** discuss strategies for handling the decision. |

Indicator 13 refers to the competency to counsel the patient regarding decision strategy. This aspect should be considered separately from the content of the consultation. In addition to providing information and exploring the patient’s preferences the doctor can also act as a coach, involving and supporting his patient in the decision-making process. Which implicit strategy does the patient follow? Is the patient satisfied with this strategy? What are the patient’s individual barriers to making this (or indeed any) decision? Which decision-making procedure has proved useful to the patient in his life so far? The patient can also initiate the topic of decision management himself by revealing his own strategy or by asking pertinent questions.

- If the topic of decision-making strategy is picked up by giving a piece of advice the competency is to be considered rather limited.
- A basic competency can be indicated by providing reasons for or suggesting a variety of possible strategies.
- Thorough examination of different strategies whilst simultaneously paying heed to the patient’s individual situation should be rated as a high level of competency, e.g. “You can base your decision on side-effects: Where will I have the least side-effects? Or on efficacy: Where might I benefit most? And there’s a third option. You can consider which administration form you prefer. Would you like to decide on that criterion?” or “Have you had to take any other important decisions in your life? How did you come to a decision there? And what helped you?“

## This kind of patient involvement should not be confused with recommendation of therapy or diagnostic protocols (medical content). Behaviour that is observed before the decision (or deferral) is made can be coded under Indicator 13

## .

| **0** | **The behaviour is not observed:**  Decision management strategies are not a topic. |
| --- | --- |
| **1** | **The behaviour is observed (minimal attempt):**  A possible strategy is suggested. This can also be achieved by asking the patient appropriate questions.  e.g. Clinician: “You seem undecided now – Am I right? This shouldn’t be something you decide in haste. Perhaps you should sleep on it.“  e.g. Patient: “I always discuss this sort of thing with my family.“ |
| **2** | **The basic competency is observed:**  Different strategies are discussed (free from medical content). Justification or explanation is provided to support appraisal of decision-making strategies.  e.g. Clinician: “One question you have to ask yourself is whether you want to have medication at all. And only when you’ve formed an opinion on that is it time to consider which option is best for you. This has proved to be a good way to proceed.“  e.g. Patient: “Hearing the opinion of my loved ones helps me come to a decision about what I really want to do.“ |
| **3** | **The behaviour is observed (good standard):**  In addition to point 2, possible decision-making strategies are discussed under consideration of the patient’s individual situation.  e.g. Clinician: “You can base your decision on side-effects: Where will I have the least side effects? Or on efficacy: Where might I benefit most? And there’s a third option. You can consider which administration form you prefer. Would you like to decide on that criterion?”  e.g. Patient: “At first I always looked to see what the side-effects were but in the meantime it’s become clear to me that my mobility is more important to me than anything else. So now it’s the possible benefit that I’m going to be looking at. Everything else comes second to that.“ |
| **4** | **The behaviour is observed (excellent standard):**  In addition to point 3, more attention is paid and more emphasis placed on the discussion of decision-making strategies.  e.g. Clinician: ”Have you had to take any other important decisions in your life? How did you come to a decision there? And what helped you?“  e.g. Patient: “I’m the sort of person who likes to gather a lot of information first. And then later on it’s my gut feeling that makes me decide one way or the other. But in this case I’m not sure whether that’s responsible.“ |

***Indicator 14:* Indicate decision**

| The **clinician** opens the decision stage leading to the selection of an option (*If applicable, deferment is a possible decision*). |
| --- |
| The **patient** opens the decision stage leading to the selection of an option (*If applicable, deferment is a possible decision*). |
| **Clinician and patient** open the decision stage leading to the selection of an option (*If applicable, deferment is a possible decision*). |

This behavioural skill indicates the transition from the stage of information exchange and negotiation to the decision stage. The competency demanded from the parties is to make this transition explicit. This is particularly important if the decision is to be deferred, for example due to a lack of clear information.

- A reliable cue for the score ”0“ is if the observer is not sure until the end of the consultation which decision has been made. It is not sufficient to open the decision stage by a brief imprecise indication. The idea is to emphasize the moment of transition in the process as clearly as possible.
- Depending on whether there have at that point of the consultation already been signs of the direction in which the decision will go and has perhaps implicitly already been taken, or whether the decision is still completely open, Indicator 14 can rate different behaviours. In the first case the idea is to explicitly agree on what has already been implicitly communicated.
- In the second case it might be necessary to summarize the relevant content of the previous information and negotiation process and thereby initiate the decision. Example “… OK, in your individual case we are talking about a remaining set of three options: drug B, which you already know, drug A, the one you could take as a tablet, or waiting to see if your condition worsens and then perhaps starting with treatment later on. Are you already able to say what you would prefer? We can also defer this decision for a week or two if you need more time“.
- Often an explicit attempt to move into the decision stage leads to renewed entry into the information process, where remaining aspects are clarified that had not been fully understood. This implies that the introduction to the decision stage has to be repeated. High competency is indicated when the transition is combined with a clear summary of the remaining options or – if there was an implicit choice already – this choice is now stated in a clearly explicit way.

***To get a higher score than “2” the list of the remaining options should always include the option to defer the decision.***

| **0** | **The behaviour is not observed:**  The parties enter the stage of volition (concrete plan of action) without any transition at all.  e.g. Clinician: “Right, so you now need an appointment for the first infusion.“  e.g. Patient: “I’ll ask for an appointment next Monday for the first infusion.“ |
| --- | --- |
| **1** | **The behaviour is observed (minimal attempt):**  The decision stage is initiated by an implicit statement.  e.g. Clinician: “So, what now?“  e.g. Patient: „“OK, fine, I’ve got the picture now.“ |
| **2** | **The basic competency is observed:**  The decision stage is initiated by an explicit statement.  e.g. Clinician: “So can you already decide on any of the options?“  e.g. Patient: “All right, I’d like to try the study drug then.“ |
| **3** | **The behaviour is observed (good standard):**  In addition to point 2, a summary is provided.  e.g. Clinician: “… Well, there are three options to choose from in your case: Drug B, with its spectrum of side-effects that you already know from your own experience, drug A that is an oral drug, or to wait and see whether your condition gets worse and you might then start a therapy later on. Can you already say which option you prefer? Another option is to defer the decision for a week or two to give you more time to think it over.“  e.g. Patient: ”… If I’ve understood everything correctly all that remains for me is to accept the infusion or to do nothing. I think I’ll …“ |
| **4** | **The behaviour is observed (excellent standard):**  In addition to point 3, more attention is paid and more emphasis placed on the transition from the information to the decision stage and the transition is conducted cooperatively.  e.g. Clinician: “Can you tell me anything else that you still need for making a decision?“  e.g. Patient: “I find it very difficult to make a decision where I can be sure that I will not regret it later on.“ |

***Indicator 15:* Follow up arrangements**

| The **clinician** makes arrangements with the patient concerning how to proceed (*e. g. steps for implementing the decision, review of decision or of deferment*). |
| --- |
| The **patient** contributes towards the arrangements for how to proceed (*e. g. steps for implementing the decision, review of decision or of deferment*). |
| **Clinician and patient** discuss plans for how to proceed (*e. g. steps for implementing the decision, review of decision or of deferment*). |

This indicator refers to aspects that have to be discussed and agreed on after a decision has been made. Again, it has to be considered that deferring a decision is also one of the possible choices. Firstly, agreements have to be made on how to implement the choice: Where should any letters/ information be sent (doctor’s report). When should the treatment begin? What diagnostic tests still have to be carried out prior to starting treatment?. Secondly, the parties should agree on how the decision will be evaluated: dates, conditions, extent and goals of check ups as well as criteria that would indicate the necessity to re-evaluate the decision.

- A superficial reference to a future consultation is not sufficient. Instead, at least a concrete agreement has to be made.
- A high level of this competency is indicated when the evaluation steps are thoroughly explained. Also, consideration of the individual patient’s daily life conditions in the further planning should be awarded higher scores, e.g.: ”Bearing your work in mind, I would suggest evaluating the treatment in terms of mobility. If this worsens, however, we should think about changing the treatment.“

***It is important that these aspects are communicated after the decision or the agreement to defer the decision. This means that if any discussion of this kind (realization or evaluation of the decision) has been carried out before the decision these aspects have to be assigned to the information process and rated accordingly by coding for Indicator 6, for example.***

| **0** | **The behaviour is not observed:**  There is no discussion of future procedure.  e.g. Clinician: “OK, then I wish you all the best with your choice of therapy.“  e.g. Patient: “Good, thank you for your advice.“ |
| --- | --- |
| **1** | **The behaviour is observed (minimal attempt):**  There is only implicit communication of future procedure (e.g.) by mentioning the need for further steps.  e.g. Clinician: “I’ll write to your GP and then you can start.“  e.g. Patient: “Where can I get the MRT images?“ |
| **2** | **The basic competency is observed:**  Future procedure is explicitly made a subject by agreeing on a follow up check up.  e.g. Clinician: “I’ll inform your GP about our consultation and your decision. And then you can arrange with him when to start treatment. And I will see you again in six month.“  e.g. Patient: “And how do we proceed from here? Do I have to see my GP first and when do we meet again?“ |
| **3** | **The behaviour is observed (good standard):**  In addition to point 2, the need for another appointment is indicated.  e.g. Clinician: “I would like you to have another MRT done so that it will be possible to track your disease course.“  e.g. Patient: “And how do I know whether I should see you again before the year’s up?“ |
| **4** | **The behaviour is observed (excellent standard):**  In addition to point 3, communication on follow-up arrangements is given more attention and individual considerations are included in the plans for further procedure.  e.g. Clinician: “ Bearing your work in mind, I would suggest evaluating the treatment in terms of mobility. If this worsens, however, we should think about changing the treatment.“  e.g. Patient: “I’d like to have an MRT done so that after one year, in addition to how I feel, and how my symptoms have developed, it’s possible to discuss my disease course on the basis of how the lesions in my brain have developed.“ |

References

Bunge M, Mühlhauser I, Steckelberg A (2010) What constitutes evidence-based patient information? Overview of discussed criteria Patient Education and Counselling 78: 316-328

Charles C, Gafni A, Whelan T (1997) Shared decision-making in the medical encounter: what does it mean? (Or it takes at least two to tango). Soc Sci Med 44: 681-692

Charles C, Gafni A, Whelan T (1999) Decision-making in the physician-patient encounter: revisiting the shared treatment decision-making model. Social Science and Medicine 49: 651-661

Elwyn G, Edwards A, Kinnersley P (1999) Shared decision-making in primary care: the neglected second half of the consultation. British Journal of General Practice 49(443): 477-482

Elwyn G, Edwards A, Mowle S, Wensing M, Wilkinson C, Kinnersley P, Grol R (2001) Measuring the involvement of patients in shared decision-making: a systematic review of instruments. Pat Educ Couns 43(1): 5-22

Elwyn G, Edwards A, Wensing, M, Hood K, Atwell C, Grol R (2003) Shared decision-making: developing the OPTION scale for measuring patient involvement. Quality and Safety in Health Care 12: 93-99

Elwyn G, Hutchings H, Edwards A, Rapport F, Wensing M, Cheung WY, and Grol R (2005) The OPTION scale: measuring the extent that clinicians involve patients in decision-making tasks. Health Expect 8: 34-42

Kasper J, Burish G, Geiger F, Heesen C (2007) Do patients perceive the involvement we observe? 4th International Shared Decision-making Conference May 30th – June 1st, 2007, Freiburg

Kasper J (2010) Evidenzbasierte Patienteninformation In: Lehrbuch der Versorgungsforschung Pfaff H, Schrappe M. Schattauer Verlag 58-63

Kasper J, Légré F, Scheibler F, Geiger F (2011) Turning signals into meaning – ‘Shared decision-making’ meets communication theory. Health Expectations; doi:10.111

Kiesler DJ, and Auerbach S (2006) Optimal matches of patient preferences for information, decision-making and interpersonal behaviour: Evidence, models and interventions. Patient Educ Couns 61: 319-341

Legaré F, Moher D, Elwyn G, LeBlanc A, Gravel K (2007) Instruments to assess the perception of physicians in the decision-making process of specific clinical encounters: a systematic review. BMC Medical Informatics and Decision Making 15:7-30.

Loh A, Simon D, Hennig K, Hennig B, Härter M, Elwyn G (2006) The assessment of depressive patients' involvement in decision-making in audio-taped primary care consultations. Patient Educ Couns 63(3): 314-8

Makoul G, Clayman M (2006) An integrative model of shared decision-making in medical encounters. Patient Educ Couns 60(3): 301

Saba GW, Wong ST, Schillinger D, Fermandez A, Somkin CP, Wilson CC, Grumbach K (2006) Shared Decision-making and the Experience of Partnership in Primary Care. Annals of Family Medicine 4(1): 54-62

Siminoff LA, Step MM (2005) A Communication Model of shared decision-making: Accounting for cancer treatment decisions. Health Psychology 24 (4): 99-105

Wirtz M, Caspar F (2002) Methoden zur Bestimmung und Verbesserung der Zuverlässigkeit von Einschätzungen mittels Kategoriensystemen und Ratingskalen. Hogrefe-Verlag

**Attachments**

- **MAPPIN’SDM observation sheet**
- **Results for reliability**
- **Comparison of OPTION and MAPPIN**

## MAPPIN’SDM questionnaire for doctor and patient (version including 15 result items = Foci 5 & 7 see p.8)

| **1** | The **clinician** draws attention to an identified problem as one that requires a decision-making process.  **MAPPIN’SDM observation sheet (Obs_doctor, patient_, _dyad_)** | 0 | 1 | 2 | 3 | 4 |
| --- | --- | --- | --- | --- | --- | --- |
|  | The **patient** draws attention to his concrete problem as one that requires a decision-making process. | 0 | 1 | 2 | 3 | 4 |
|  | **Clinician and patient** agree on a concrete problem as one that requires a decision-making process. | 0 | 1 | 2 | 3 | 4 |

| **2** | The **clinician** states that there is more than one way to deal with the identified problem (‘*equipoise*’). | 0 | 1 | 2 | 3 | 4 |
| --- | --- | --- | --- | --- | --- | --- |
|  | The **patient** indicates that there is more than one way to deal with the concrete problem (‘*equipoise*’). | 0 | 1 | 2 | 3 | 4 |
|  | **Clinician and patient** discuss that there is more than one way to deal with the concrete problem (‘*equipoise*’). | 0 | 1 | 2 | 3 | 4 |

| **3** | The **clinician** ascertains the patient’s preferred approach to exchanging information (*e.g. in which setting, with which media, which time frame*). | 0 | 1 | 2 | 3 | 4 |
| --- | --- | --- | --- | --- | --- | --- |
|  | The **patient** participates in deciding on the preferred approach to exchanging information (*e.g. in which setting, with which media, which time frame*). | 0 | 1 | 2 | 3 | 4 |
|  | **Clinician and patient** choose an approach to exchanging information (*e.g. in which setting, with which media, which time frame*). | 0 | 1 | 2 | 3 | 4 |

| **4** | The **clinician** elicits the patient’s preferred level of involvement in decision-making. | 0 | 1 | 2 | 3 | 4 |
| --- | --- | --- | --- | --- | --- | --- |
|  | The **patient** expresses his preferred level of involvement in decision-making. | 0 | 1 | 2 | 3 | 4 |
|  | **Clinician and patient** discuss role distribution during the consultation. | 0 | 1 | 2 | 3 | 4 |

| **5** | The **clinician** lists the options (*If ‘doing nothing / deferring the decision’ is possible, this option should be included in the list*). | 0 | 1 | 2 | 3 | 4 |
| --- | --- | --- | --- | --- | --- | --- |
|  | The **patient** lists the options (*If ‘doing nothing / deferring the decision’ is possible, this option should be included in the list*). | 0 | 1 | 2 | 3 | 4 |
|  | **Clinician and patient** list the options (*If ‘doing nothing / deferring the decision’ is possible, this option should be included in the list*). | 0 | 1 | 2 | 3 | 4 |

**MAPPIN’SDM observation sheet**

| **6** | The **clinician** explains to the patient the pros and cons of the different options (*if applicable, also the pros and cons of ‘doing nothing’*). | 0 | 1 | 2 | 3 | 4 |
| --- | --- | --- | --- | --- | --- | --- |
|  | The **patient** discusses the pros and cons of the different options (*if applicable also the pros and cons of ‘doing nothing’*). | 0 | 1 | 2 | 3 | 4 |
|  | **Clinician and patient** weigh up the pros and cons of the different options (*if applicable, also the pros and cons of ‘doing nothing’*). | 0 | 1 | 2 | 3 | 4 |

| **7** | The **clinician** explores the patient’s expectations (*ideas*) and concerns (*fears*) about how to manage the concrete problem. | 0 | 1 | 2 | 3 | 4 |
| --- | --- | --- | --- | --- | --- | --- |
|  | The **patient** describes his expectations (*ideas*) and concerns (*fears*) about how to manage the concrete problem. | 0 | 1 | 2 | 3 | 4 |
|  | **Clinician and patient** discuss the patient’s expectations (*ideas*) and concerns (*fears*) about how to manage the concrete problem. | 0 | 1 | 2 | 3 | 4 |

| **8** | The **clinician** makes known to the patient the source upon which his information / recommendations are based (*scientific evidence, own judgement, preferences, conflicting interests*). | 0 | 1 | 2 | 3 | 4 |
| --- | --- | --- | --- | --- | --- | --- |
|  | The **patient** clarifies the source upon which medical information / recommendations are based (*scientific evidence, clinician’s judgement, preferences, conflicting interests*). | 0 | 1 | 2 | 3 | 4 |
|  | **Clinician and patient** clarify the source upon which medical information / recommendations are based (*scientific evidence, clinician’s judgement, preferences, conflicting interests*). | 0 | 1 | 2 | 3 | 4 |

| **9** | The **clinician** checks that the patient has understood the information. | 0 | 1 | 2 | 3 | 4 |
| --- | --- | --- | --- | --- | --- | --- |
|  | The **patient** clarifies how he understood the information given by the clinician. | 0 | 1 | 2 | 3 | 4 |
|  | **Clinician and patient** clarify whether the patient understood the information given by the clinician correctly. | 0 | 1 | 2 | 3 | 4 |

| **10** | The **clinician** makes sure that he has understood the patient’s viewpoint correctly. | 0 | 1 | 2 | 3 | 4 |
| --- | --- | --- | --- | --- | --- | --- |
|  | The **patient** makes sure that the clinician understands his viewpoint. | 0 | 1 | 2 | 3 | 4 |
|  | **Clinician and patient** clarify whether the clinician has understood the patient’s viewpoint correctly. | 0 | 1 | 2 | 3 | 4 |

## MAPPIN’SDM observation sheet

| **11** | The **clinician** explicitly offers the patient opportunities to ask questions and to point out aspects he had not fully understood during the discussion. | 0 | 1 | 2 | 3 | 4 |
| --- | --- | --- | --- | --- | --- | --- |
|  | The **patient** asks questions or points out aspects he had not fully understood during the discussion. | 0 | 1 | 2 | 3 | 4 |
|  | **Clinician and patient** make sure that the patient can ask questions and point out aspects he had not fully understood during the discussion. | 0 | 1 | 2 | 3 | 4 |

| **12** | The **clinician** asks questions or points out aspects he had not fully understood during the discussion. | 0 | 1 | 2 | 3 | 4 |
| --- | --- | --- | --- | --- | --- | --- |
|  | The **patient** explicitly offers the clinician opportunities to ask questions or to point out aspects he had not fully understood during the discussion. | 0 | 1 | 2 | 3 | 4 |
|  | **Clinician and patient** make sure that the clinician can ask questions and point out aspects he had not fully understood during the discussion.. | 0 | 1 | 2 | 3 | 4 |

| **13** | The **clinician** supports the patient in his activation of decision-making strategies. | 0 | 1 | 2 | 3 | 4 |
| --- | --- | --- | --- | --- | --- | --- |
|  | The **patient** talks about his decision-making strategies. | 0 | 1 | 2 | 3 | 4 |
|  | **Clinician and patient** discuss strategies for handling the decision. | 0 | 1 | 2 | 3 | 4 |

| **14** | The **clinician** opens the decision stage leading to the selection of an option (*If applicable, deferment is a possible decision*). | 0 | 1 | 2 | 3 | 4 |
| --- | --- | --- | --- | --- | --- | --- |
|  | The **patient** opens the decision stage leading to the selection of an option (*If applicable, deferment is a possible decision*). | 0 | 1 | 2 | 3 | 4 |
|  | **Clinician and patient** open the decision stage leading to the selection of an option (*If applicable, deferment is a possible decision*). | 0 | 1 | 2 | 3 | 4 |

| **15** | The **clinician** makes arrangements with the patient concerning how to proceed (*e.g. steps for implementing the decision, review of decision or of deferment*). | 0 | 1 | 2 | 3 | 4 |
| --- | --- | --- | --- | --- | --- | --- |
|  | The **patient** contributes towards the arrangements for how to proceed (*e.g. steps for implementing the decision, review of decision or of deferment*). | 0 | 1 | 2 | 3 | 4 |
|  | **Clinician and patient** discuss plans for how to proceed (*e.g. steps for implementing the decision, review of decision or of deferment*). | 0 | 1 | 2 | 3 | 4 |

**Results for reliability**

|  | Obs_doctor_ | | | | Obs_patient_ | | | | Obs_dyad_ | | | |
| --- | --- | --- | --- | --- | --- | --- | --- | --- | --- | --- | --- | --- |
|  | Mean | SD^A^ | Agree.^B^ | Reliability^C^ | Mean | SD^A^ | Agree.^B^ | Reliability^C^ | Mean | SD^A^ | Agree.^B^ | Reliability^C^ |
| **1** | 1,04 | 0,98 | 93.6 | .90 | 0,19 | 0,54 | 89.4 | .62 | 1,13 | 1,01 | 80.9 | .90 |
| **2** | 0,11 | 0,38 | 95.7 | .86 | 0,11 | 0,38 | 93.6 | .55 | 0,19 | 0,50 | 87.5 | .71 |
| **3** | .043 | .29 | 97.9 | 1 | .04 | .29 | 97.9 | 1 | .04 | .29 | 100 | 1 |
| **4** | .85 | .59 | 83 | .71 | .64 | .79 | 80.9 | .85 | .98 | .71 | 91.5 | .91 |
| **5** | .68 | .88 | 83 | .89 | .08 | .28 | 95.8 | .69 | .69 | .88 | 81.3 | .88 |
| **6** | 1.21 | .74 | 70.8 | .68 | .54 | .65 | 68.3 | .55 | 1.33 | .81 | 72.9 | .68 |
| **7** | 1.77 | .63 | 48.9 | .32 | 2.53 | .91 | 51.1 | .61 | 2.60 | .88 | 48.9 | .51 |
| **8** | .87 | .88 | 70.2 | .80 | .11 | .43 | 95.7 | .86 | .87 | .90 | 68.1 | .78 |
| **9** | .00 | .00 | 97.9 | - | .09 | .41 | 97.9 | 1 | .09 | .41 | 97.9 | .94 |
| **10** | ,60 | ,93 | 78.7 | .65 | ,04 | ,29 | 97.9 | - | ,62 | ,97 | 76.6 | .66 |
| **11** | 1,21 | ,59 | 87.2 | .74 | 1,43 | 1,25 | 76.6 | .80 | 1,92 | ,86 | 76.6 | .71 |
| **12** | ,23 | ,63 | 89.4 | .57 | ,00 | ,00 | 100 | - | ,23 | ,63 | 89.4 | .57 |
| **13** | ,23 | ,60 | 74.5 | .38 | ,15 | ,47 | 87.2 | .43 | ,34 | ,67 | 66 | .34 |
| **14** | 1,53 | ,93 | 59.6 | .69 | 1,04 | 1 | 63.8 | .52 | 2,04 | ,86 | 51.1 | .30 |
| **15** | 1,96 | 1,03 | 65.2 | .79 | 1,37 | 1,08 | 50 | .59 | 2,00 | 1,03 | 65.2 | .81 |
| **Item-level**  **Mean(15)** | 0.82 | 0.67 | 79.7 | .77 | 0.56 | 0.59 | 83.1 | .70 | 1.01 | 0.76 | 76.9 | .71 |
| **Item-level**  **Mean(12)** |  |  | 80.1 | .76 |  |  |  | . |  |  |  |  |
| **Mean level (15)** |  |  |  | .90 |  |  |  | .85 |  |  |  | .91 |

The table shows the inter-rater reliability (pair wise average) of the relevant rater pairs out of a group of four raters applying MAPPIN’SDM to a sample of 50 video-taped consultations. All raters had previously taken part in training with 27 videos. Pairs rotated systematically.

A= calculated based on consensus ratings for the same sample. Range: 0- 4.

B= percentage agreement

C= Pearson correlation coefficient (Values calculated with Spearman’s rho are quite similar)

**Comparison of OPTION and MAPPIN’SDM**

| **SDM aspect** | **OPTION** | **MAPPIN’SDM** |
| --- | --- | --- |
| defining problem | 1 | 1 |
| equipoise statement | 2 | 2 |
| preferred communication approach | 3 | 3 |
| listing options | 4 | 5 |
| pros & cons | 5 | 6 |
| expectations | 6 | 7 |
| worries | 7 |
| indicating source of recommendations/evidence |  | 8 |
| doctor’s evaluation of patient’s understanding | 8 | 9 |
| patient’s evaluation of physician’s understanding |  | 10 |
| opportunity for questions (from patient) | 9 | 11 |
| opportunity for questions (from physician) |  | 12 |
| role attribution | 10 | 4 |
| supporting strategies of decision-making |  | 13 |
| indicate decision | 11 | 14 |
| follow up arrangements | 12 | 15 |

MAPPIN’SDM questionnaire

| **Doctor and patient questionnaire for assessing consultations** | | | | | |
| --- | --- | --- | --- | --- | --- |
| Dear Doctor,  This questionnaire addresses both parties of a doctor-patient consultation about a medical decision (e.g. concerning treatment or examination). The aim is to ascertain to what extent both parties perceive the consultation in a similar way. It is thus important that both parties answer all questions independently of each other and directly after the consultation. | | | | | |
| Please read all the questions before the consultation begins! | | | | | |
| Apart from the reference, the questions are identical for doctor and patient, e.g.:  *“The patient understood the information…”* or accordingly:  *“I understood the information …”* | | | | | |
| Please mark the score ( ) that you consider most applicable. |  | | | | |
|  |  |  |  |  |  |
|  | **not at all** | |  | **absolutely true** | |
|  | 0 | 1 | 2 | 3 | 4 |
| Your answers will not be shown to the other party!! | | | | | |
| Before you separate please agree on which decision(s) you talked about and fill in together which decision you will think about when filling in the questionnaire:(Please select only one) | | | | | |
| Please enter here: e.g. “Whether to start immunotherapy” or “Which prenatal tests” | (z. B.“Frage ob Aufnahme einer Immuntherapie“  oder „welche vorgeburtlichen Untersuchungen“) | | | | |
| Important: You should both be referring to the same decision. | | | | | |
|  |  | Thank you for completing this questionnaire! | | | |

| **1** The medical problem that requires a decision-making process is clear to the patient. | **not at all** | |  | **absolutely true** | |
| --- | --- | --- | --- | --- | --- |
|  | **0** | **1** | **2** | **3** | **4** |

| **2** I am convinced that from a medical point of view there is not only one correct way to deal with this problem. Several basically equivalent ways are conceivable. The patient first has to clarify which of the respective pros and cons are important for him personally. It’s not possible for me as the doctor to know which option is the right one in this case. | **not at all** | |  | **absolutely true** | |
| --- | --- | --- | --- | --- | --- |
|  | **0** | **1** | **2** | **3** | **4** |

| **3** The way I exchanged information with the patient during the consultation suited both parties and contributed towards a mutual understanding (*e.g. setting, verbal or graphic information*). | **not at all** | |  | **absolutely true** | |
| --- | --- | --- | --- | --- | --- |
|  | **0** | **1** | **2** | **3** | **4** |

| **4** Role distribution during the consultation matched the patient’s wishes (*meaning: ‘balance of power’, distribution of responsibilities in the decision-making process*). | **not at all** | |  | **absolutely true** | |
| --- | --- | --- | --- | --- | --- |
|  | **0** | **1** | **2** | **3** | **4** |

| **5** The patient is aware of all the options for dealing with his current problem (*If applicable including that of doing without examination or treatment*). | **not at all** | |  | **absolutely true** | |
| --- | --- | --- | --- | --- | --- |
|  | **0** | **1** | **2** | **3** | **4** |

| **6** The patient now knows the pros and cons of the different decision options (*If applicable, also the pros and cons of the option to do without an examination and treatment*). | **not at all** | |  | **absolutely true** | |
| --- | --- | --- | --- | --- | --- |
|  | **0** | **1** | **2** | **3** | **4** |

| **7** The patient’s personal expectations and fears went into the decision. | **not at all** | |  | **absolutely true** | |
| --- | --- | --- | --- | --- | --- |
|  | **0** | **1** | **2** | **3** | **4** |

| **8** It became clear to the patient what my medical information and recommendations are based on (*scientific evidence, my own judgement, benefits that I myself have when a certain measure is chosen, e.g. commission / research interests*). | **not at all** | |  | **absolutely true** | |
| --- | --- | --- | --- | --- | --- |
|  | **0** | **1** | **2** | **3** | **4** |

| **9** The patient understood the information that I gave him. | **not at all** | |  | **absolutely true** | |
| --- | --- | --- | --- | --- | --- |
|  | **0** | **1** | **2** | **3** | **4** |

| **10** I understood the patient’s viewpoint. | **not at all** | |  | **absolutely true** | |
| --- | --- | --- | --- | --- | --- |
|  | **0** | **1** | **2** | **3** | **4** |

| **11** The patient cleared up the questions and aspects he had not fully understood during the discussion. | **not at all** | |  | **absolutely true** | |
| --- | --- | --- | --- | --- | --- |
|  | **0** | **1** | **2** | **3** | **4** |

| **12** I cleared up the questions and aspects I had not fully understood during the discussion. | **not at all** | |  | **absolutely true** | |
| --- | --- | --- | --- | --- | --- |
|  | **0** | **1** | **2** | **3** | **4** |

| **13** It has become clear to the patient what his decision-making strategy will be  (i. e. how the patient will proceed when he makes the decision). | **not at all** | |  | **absolutely true** | |
| --- | --- | --- | --- | --- | --- |
|  | **0** | **1** | **2** | **3** | **4** |

| **14** At the end of the consultation it was clear to the patient why and which decision was taken (*If appropriate, the decision could be ‘to defer’*). | **not at all** | |  | **absolutely true** | |
| --- | --- | --- | --- | --- | --- |
|  | **0** | **1** | **2** | **3** | **4** |

| **15** It is now clear to the patient how his problem will in future be dealt with (*e.g. who has to inform whom; when the two of us will review the decision or the deferment*). | **not at all** | |  | **absolutely true** | |
| --- | --- | --- | --- | --- | --- |
|  | **0** | **1** | **2** | **3** | **4** |

| **Doctor and patient questionnaire for assessing consultations** | | | | | | | | | |
| --- | --- | --- | --- | --- | --- | --- | --- | --- | --- |
| Dear Patient,  This questionnaire addresses both parties of a doctor-patient consultation about a medical decision (e.g. concerning treatment or examination). The aim is to ascertain to what extent both parties perceive the consultation in a similar way. It is thus important that both parties answer all questions independently of each other and directly after the consultation. | | | | | | | | | |
| Please read all the questions before the consultation begins! | | | | | | | | | |
| Apart from the reference, the questions are identical for doctor and patient, e.g.  *“The patient understood the information…”* or correspondingly:  *“I understood the information… ”* | | | | | | | | | |
| Please mark the score ( )  that you consider most applicable. | |  | | | | | | | |
|  |  |  |  |  |  |  |  |  |  |
|  |  | **not at all** | | |  | | | **absolutely true** | |
|  |  | 0 | | 1 | | 2 | 3 | | 4 |
| Your answers will not be shown to the other party!! | | | | | | | | | |
| Before you separate please agree on which decision(s) you talked about and fill in together which decision you will think about when filling in the questionnaire:(Please select only one) | | | | | | | | | |
| Please enter here: e.g. “Whether to start immunotherapy” or “Which prenatal tests” | |  | | | | | | | |
| Important: You should both be referring to the same decision. | | | | | | | | | |
|  |  | | Thank you for completing this questionnaire! | | | | | | |

| **1** The concrete medical problem that requires a decision-making process is clear to me. | **not at all** | |  | **absolutely true** | |
| --- | --- | --- | --- | --- | --- |
|  | **0** | **1** | **2** | **3** | **4** |

| **2** I am convinced that from a medical point of view there is not only one correct way to deal with my problem. Several basically equivalent ways are conceivable. As a patient I first have to clarify which of the respective pros and cons are important for me personally. The doctor cannot decide on his own which option is the right one in my case. | **not at all** | |  | **absolutely true** | |
| --- | --- | --- | --- | --- | --- |
|  | **0** | **1** | **2** | **3** | **4** |

| **3** The way I exchanged information with the doctor during the consultation suited both parties and contributed towards a mutual understanding (*e.g. setting, verbal or graphic information*). | **not at all** | |  | **absolutely true** | |
| --- | --- | --- | --- | --- | --- |
|  | **0** | **1** | **2** | **3** | **4** |

| **4** Role distribution during the consultation matched my wishes (*meaning: ‘balance of power’, distribution of responsibilities in the decision-making process*). | **not at all** | |  | **absolutely true** | |
| --- | --- | --- | --- | --- | --- |
|  | **0** | **1** | **2** | **3** | **4** |

| **5** I am aware of all the options for dealing with my current problem (*If applicable including that of doing without examination or treatment*). | **not at all** | |  | **absolutely true** | |
| --- | --- | --- | --- | --- | --- |
|  | **0** | **1** | **2** | **3** | **4** |

| **6** I now know the pros and cons of the different decision options (I*f applicable, also the pros and cons of the option to do without an examination and treatment*). | **not at all** | |  | **absolutely true** | |
| --- | --- | --- | --- | --- | --- |
|  | **0** | **1** | **2** | **3** | **4** |

| **7** My personal expectations and fears went into the decision. | **not at all** | |  | **absolutely true** | |
| --- | --- | --- | --- | --- | --- |
|  | **0** | **1** | **2** | **3** | **4** |

| **8** It became clear to me what the medical information and recommendations are based on (*scientific evidence, doctor’s judgement, benefits that the doctor has if a certain measure is chosen, e.g. commission, research interests*). | **not at all** | |  | **absolutely true** | |
| --- | --- | --- | --- | --- | --- |
|  | **0** | **1** | **2** | **3** | **4** |

| **9** I understood the information the doctor gave me. | **not at all** | |  | **absolutely true** | |
| --- | --- | --- | --- | --- | --- |
|  | **0** | **1** | **2** | **3** | **4** |

| **10** The doctor understood my viewpoint. | **not at all** | |  | **absolutely true** | |
| --- | --- | --- | --- | --- | --- |
|  | **0** | **1** | **2** | **3** | **4** |

| **11** I cleared up the questions and aspects I had not fully understood during the discussion. | **not at all** | |  | **absolutely true** | |
| --- | --- | --- | --- | --- | --- |
|  | **0** | **1** | **2** | **3** | **4** |

| **12** The doctor cleared up the questions and aspects he had not fully understood during the discussion. | **not at all** | |  | **absolutely true** | |
| --- | --- | --- | --- | --- | --- |
|  | **0** | **1** | **2** | **3** | **4** |

| **13** My decision-making strategy has become clear to me (*i.e. how I will proceed when I make the decision*). | **not at all** | |  | **absolutely true** | |
| --- | --- | --- | --- | --- | --- |
|  | **0** | **1** | **2** | **3** | **4** |

| **14** At the end of the consultation it was clear to me why and which decision was taken (*If appropriate, the decision could be ‘to defer’*). | **not at all** | |  | **absolutely true** | |
| --- | --- | --- | --- | --- | --- |
|  | **0** | **1** | **2** | **3** | **4** |

| **15** It is now clear to me how my problem will in future be dealt with (*e.g. who has to inform whom; when the two of us will review the decision or the deferment*). | **not at all** | |  | **absolutely true** | |
| --- | --- | --- | --- | --- | --- |
|  | **0** | **1** | **2** | **3** | **4** |
